# Hypersensitivity in Orthodontics: A Systematic Review of Oral and Extra-Oral Reactions

**DOI:** 10.3390/jcm14134766

**Published:** 2025-07-05

**Authors:** Alessandra Amato, Stefano Martina, Giuseppina De Benedetto, Ambrosina Michelotti, Massimo Amato, Federica Di Spirito

**Affiliations:** 1 Department of Neuroscience, Reproductive Science and Dentistry, University of Naples Federico II, 80131 Naples, Italy; aaleamato@gmail.com (A.A.); ambrosina.michelotti@unina.it (A.M.); 2 Department of Medicine, Surgery and Dentistry, University of Salerno, Via S. Allende, 84081 Baronissi, Italy; smartina@unisa.it (S.M.); giusydb15@gmail.com (G.D.B.)

**Keywords:** orthodontics, orthodontic treatment, orthodontic appliance, hypersensitivity, hypersensitivity reactions, oral lesions, nickel, titanium, resins

## Abstract

**Background/Objectives**: This systematic review analyzed the epidemiologic and macro/microscopic features of manifestations of hypersensitivity reactions with oral and extra-oral involvement in orthodontic patients with fixed (FAs) or removable (RAs) appliances or clear aligners (CAs), and evaluated them based on patient and treatment characteristics to provide clinical recommendations. **Methods**: The study protocol followed the PRISMA guidelines and was registered on PROSPERO (CRD42024517942). **Results**: Thirty-one studies were qualitatively assessed and synthetized, involving 858 subjects (114 males and 714 females, 9–49 years old), of whom there were 86 with a history of allergy, and 743 wearing recorded appliances (FAs = 656, FAs and RAs = 81, intra- and extra-oral RAs = 3, CAs = 3), with a mean treatment duration of 21.5 months (6 weeks–40 months). Among 75 reports, 29 (38.67%), describing burning, gingival hyperplasia, erythema, and vesicles, had oral involvement, while 46 (61.33%) had skin, eye, and systemic involvement, with erythema, papules, conjunctival hyperemia, and vertigo. Positive allergy tests concomitant with the manifestations identified nickel 451 times, cobalt 6 times, titanium 5 times, and chromium 4 times. Management included antihistamines or corticosteroids and removing the offending materials, with treatment discontinuation/appliance substitution. **Conclusions**: Pre-treatment evaluations, including patient histories and allergy testing, are essential to identify potential allergens and select hypoallergenic materials like titanium or ceramic brackets; regular monitoring and early intervention during treatment are crucial to prevent severe outcomes.

## 1. Introduction

Oral and extra-oral (both perioral and systemic) manifestations of hypersensitivity reactions are adverse inflammatory responses triggered by allergens present in dental materials, particularly polymers and metals [1]. These reactions occur when the immune system overreacts to specific allergens found in dental materials [2].

From a clinical perspective, oral manifestations related to hypersensitivity reactions can present variably but commonly involve erythema, gingival hyperplasia, and erosive-ulcerative lesions, often accompanied by itching, pain, and burning sensations [3,4], and can pose significant challenges [5].

Among the most common manifestations are oral lichenoid lesions, which can appear in proximity to dental restorations, especially amalgam, presenting as reticular, white, or erosive mucosal patches, erythema, swelling, and undefined erythematous or erosive-ulcerative manifestations, often accompanied by burning symptoms. Similar features are noted for lips, perioral area, feet, and hand lesions, with occasional systemic involvement [5,6]. Histologically, these lesions resemble other contact hypersensitivity reactions, such as the band-like infiltrate of lymphocytic cells within the superficial lamina propria, basal cell degeneration, and hyperkeratosis, aiding differential diagnosis from autoimmune or infectious disorders [7].

The most commonly implicated dental material allergens are metals, used in dental amalgams like nickel, palladium, chromium, and cobalt [5,7]. Diagnosis is based on a thorough clinical exam, a detailed patient history, and allergy tests, such as patch or prick tests, definitively confirmed when symptoms regress after the removal or replacement of the suspected material [5]. Treatment typically involves removing and replacing the offending material with biocompatible alternatives, such as composites or glass ionomer cements, often resulting in marked clinical improvement [8]. In addition, topical corticosteroids may be used to reduce inflammation and alleviate symptoms, while systemic corticosteroids or immunosuppressive therapies may be required in systemic involvement cases [8].

The progression of oral manifestations largely depends on the timely identification and removal of the allergen, as a delayed diagnosis can lead to chronic symptoms and increased morbidity [7,8,9]. Preemptive testing may be considered for subjects with a history of allergies, in particular to metals or acrylates, before extensive dental work [8].

The incidence varies widely, with estimates ranging from 1:700 to 1:2600 in dental specialized practices [5]. The female gender appears to be more commonly affected, accounting for approximately 70% of reported cases [5]. The average age of affected individuals is around 50 years, with a higher prevalence in those over 50 years of age [5].

Individuals presenting with these hypersensitivity manifestations often report a personal or family history of atopic affections (e.g., asthma/allergic rhinitis/atopic dermatitis) or allergies to metals such as nickel, cobalt, chromium, and palladium, as well as to acrylates found in removal prostheses and composites, and to other common allergens like latex and drugs [5,10]. Ongoing pharmacological treatments, especially immunosuppressive or anti-inflammatory drugs, may influence the presentation and severity of reactions [5,10].

Although the oral manifestations of hypersensitivity reactions are uncommon (0.3–0.4% of dental patients), these reactions are clinically relevant, especially among subjects with an atopy history or sensitized to metals [2]. For this reason, understanding and recognizing the clinical and histological characteristics of these manifestations, along with valuable diagnostic and treatment approaches, is essential for orthodontic practitioners and dental professionals to manage and prevent additional reactions effectively [11].

Moreover, nickel is a notable allergen in this context [2], whose hypersensitivity affects an estimated 10–30% of women and 1–3% of men [3]. In orthodontic patients, prolonged exposure to nickel-containing appliances may heighten the risk of allergic reactions [12]. Evidence suggests that up to 30% of female and 3% of male orthodontic patients may develop hypersensitivity reactions to nickel, often related to previous sensitization from non-dental sources such as earrings or body piercings [3,11]. The oral manifestations of hypersensitivity reactions in orthodontic subjects are also a relevant challenge because of their heterogeneous presentations, causing discomfort and pain that may negatively affect patients’ well-being and quality of life, necessitating careful clinical management [13]. In this context, salivary markers such as interleukin-1β have been proposed as valuable markers for monitoring periodontal status during orthodontic treatment, especially in patients with hypersensitivity or inflammatory conditions [14]. 

Therefore, the primary aim of the present systematic review was to analyze the prevalence, clinics, and microscopy of manifestations potentially related to hypersensitivity reactions with oral (mucosal lesions, hypo-/dys-geusia ± hyposmia, dysestesia) and extra-oral (skin, eye, and systemic) involvement in orthodontic subjects with (at least) one positive reaction to an allergy test before and/or during and/or after orthodontic treatment, assessing their relative frequency in patients treated with fixed or removable appliances or clear aligners.

The secondary objectives were to comprehensively evaluate reported manifestations according to patients’ age, gender, (ascertained) family and/or medical history of positive reactions to allergy tests and (any) hypersensitivity manifestations prior to orthodontic treatment, comorbidities and associated ongoing therapies, as well as orthodontic appliance, time since initiation and the total duration of orthodontic treatment, and need for interruption and/or appliance replacement, to provide clinical recommendations for managing orthodontic treatment in patients with (at least) one positive reaction to an allergy test before and/or during and/or after orthodontic treatment.

## 2. Materials and Methods

### 2.1. Study Protocol

The study protocol was developed under the Preferred Reporting Items for Systematic Reviews and Meta-analysis (PRISMA) statement [15] and registered on the PROSPERO systematic review register (registration number: CRD42024517942) before the literature search completion.

The focused research question [16] concerned the prevalence, clinical and histologic features, diagnosis, therapy, progression, time to onset, and impact on the orthodontic treatment of oral and mucocutaneous lesions, systemic manifestations, and sensory dysfunction potentially associated with hypersensitivity reactions in orthodontic subjects with (at least) one positive reaction to allergy testing before, during, or after orthodontic treatment with removable/fixed appliances or clear aligners.

**Figure 1:**
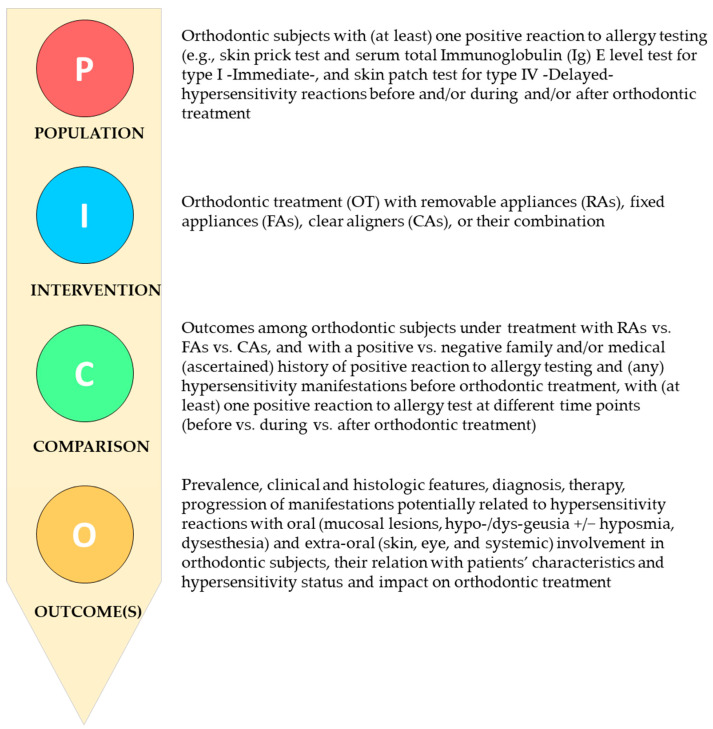
PICO model [17]: Population [18]; Intervention [19]; Comparison; Outcome(s).

The development of strategies for formulating questions, conducting searches, and selecting studies relied on the framework provided by the PICO model [17], as depicted in Figure 1.

### 2.2. Search Strategy

Case reports, case series, cross-sectional, case-control, retrospective, and prospective studies, as well as Randomized Clinical Trials (RCTs) in English language reporting or investigating hypersensitivity reactions in orthodontic subjects, were searched electronically by two reviewers (M.A. and F.D.S.) across the Scopus, MEDLINE/PubMed and Web of Science electronic databases, and PROSPERO register, until 02 January 2025, using the following keywords combined with Boolean operators: “hypersensitivity” OR “hypersensitivity reaction” OR allergy OR allergic OR “allergic reaction” OR “adverse reaction” OR sensitivity OR sensitive OR sensitivities OR sensitives AND “orthodontic appliances” OR “orthodontic material” OR “aligner” OR “Invisalign” OR “clear aligner”.

The following search filters were applied: “excluded review”, “human”, “humans”, “English”, in the Scopus database; “humans”, “English”, “exclude preprints”, in the MEDLINE/PubMed database; “excluded review article”, “article”, “English” in the Web of Science database; no filters in the PROSPERO register.

### 2.3. Study Selection and Eligibility Criteria

The collected references were managed by Mendeley Reference Manager. Duplicates were eliminated, and two independent reviewers (A.A. and F.D.S.) conducted a preliminary screening of titles and abstracts. For potentially relevant papers, or in cases of ambiguous abstracts, the full texts were obtained and the study authors were contacted when necessary; papers were reviewed independently by the same authors (A.A. and F.D.S.), who resolved disagreements through discussion and consensus and, if necessary, with the involvement of a third reviewer (M.A.).

Additionally, a manual search was also performed by screening the reference lists of articles included; relevant titles and abstracts were screened, and full texts were reviewed as described above.

Inclusion criteria were as follows: case reports, case series, cross-sectional, case-control, retrospective, and prospective studies, as well as RCTs accepted and published in the English language, without restrictions on publication date; records involving orthodontic subjects with (at least) one positive reaction to allergy testing before and/or during and/or after orthodontic treatment with no restrictions concerning sample size, participants’ age, gender, comorbidities and ongoing pharmacological therapies, history of (any) allergy; any orthodontic treatment with removable/fixed appliances or clear aligners, also discontinued or modified due to ascertained hypersensitivity to orthodontic materials; any oral and mucocutaneous lesions, systemic manifestations, and neurosensory involvement potentially related to hypersensitivity reactions.

Exclusion criteria were as follows: in vitro and animal studies, systematic and narrative reviews, pre-printed versions, and articles published in non-English languages; subjects not undergoing orthodontic treatment with removable/fixed appliances or clear aligners, and subjects with a negative reaction to or never undergone allergy testing; self-reported oral and mucocutaneous lesions, systemic manifestations, and neurosensory involvement potentially related to hypersensitivity reactions.

### 2.4. Data Extraction and Collection

Three independent reviewers (S.M., G.D.B., and A.M.) extracted data, consulting a fourth reviewer (M.A.) in cases of discrepancies.

Following models proposed for intervention reviews of RCTs and non-RCTs, a standardized data extraction form was used to collect data meeting eligibility criteria from each study. This included the following:

- Source: First author, year, journal, study type and quality, funding;
- Population: sample size, age range and mean, gender ratio, history of allergies or hypersensitivity (any), previous allergy test, other comorbidities, ongoing pharmacological therapies; piercing;
- Intervention: orthodontic treatment appliance, type of brackets, type of archwire, orthodontic material, orthodontic treatment duration, orthodontic treatment discontinuation/appliance replacement;
- Manifestation(s) potentially associated with hypersensitivity reactions with extra-oral involvement: skin involvement, eye involvement, skin location, eye involvement, systemic involvement;
- Manifestation(s) potentially associated with hypersensitivity reactions with oral involvement; oral macroscopic features, oral macroscopic features, number, distribution, location, microscopic features, time to onset;
- Diagnosis, therapy, and progression of the manifestation(s) potentially associated with hypersensitivity reactions: allergy test(s), differential diagnosis, definitive oral and extra-oral diagnosis, pharmacological therapy, treatment, resolution/progression, timing of allergic test performed, evidence of orthodontic material allergy.

### 2.5. Data Synthesis

Only data from subjects with (at least) one positive reaction to allergy testing (e.g., skin prick test and serum total Immunoglobulin (Ig) E level test for type I -Immediate-, and skin patch test for type IV -Delayed- hypersensitivity reactions [18] before and/or during and/or after orthodontic treatment involved in the studies included were qualitatively synthesized through the Microsoft Excel software 2021 (Microsoft Corporation, Redmond, WA, USA):

➢ to estimate the prevalence of manifestations potentially related to hypersensitivity reactions with oral and extra-oral (skin, eye, and systemic) involvement in orthodontic subjects with (at least) one positive reaction to allergy testing before and/or during and/or after orthodontic treatment;
➢ to assess the relative frequency in orthodontic subjects treated with fixed or removable appliances, or clear aligners;
➢ to characterize their reported macroscopic and microscopic features, time to onset, diagnosis, therapy, and progression;
➢ to evaluate them in relation to orthodontic patients’ age, gender, (ascertained) family and/or medical history of positive reaction to allergy testing and (any) hypersensitivity manifestations before orthodontic treatment, comorbidities, and related ongoing therapies;
➢ to evaluate them in relation to the orthodontic appliance and the time since the beginning and total duration of the orthodontic treatment;
➢ to assess the reported need for orthodontic treatment suspension and/or orthodontic appliance substitution.

### 2.6. Quality Assessment

In the present systematic review, the risk of bias among the included studies was assessed by three reviewers (A.M., F.D.S., A.A.) independently, through the use of the Risk of Bias Instrument for Non-randomized Studies of Exposures [20], and the Revised Cochrane risk-of-bias tool for randomized trials (RoB 2) [21], as appropriate.

## 3. Results

### 3.1. Study Selection

In total, 1101 records were originally identified, 359 from Scopus, 328 from MEDLINE/PubMed, 314 from Web of Science electronic databases, and 0 from PROSPERO register. A total of 360 duplicates were eliminated, and 641 title-abstracts were screened. A total of 586 title-abstracts were excluded, and 55 full-texts were reviewed. For three records, the full text was requested, but no response was found. A total of 52 full texts retrieved were screened, and an additional 24 articles were excluded, specifically because: they were in vitro studies (n = 8); orthodontic subjects with (at least) one positive reaction to allergy testing were not involved (n = 7); they were reviews (n = 7); self-reported lesions were involved (n = 1); it was a commentary (n = 1).

Finally, 28 studies [1,4,12,22,23,24,25,26,27,28,29,30,31,32,33,34,35,36,37,38,39,40,41,42,43,44,45,46] were included from the electronic search.

The same study selection process was conducted to identify additional studies from the manual research, screening the lists of references of the included studies by performing an electronic search.

A total of 592 references were retrieved, and 193 duplicate records were removed. The remaining 399 title-abstracts were screened and 381 were excluded because they were not relevant to the topic of the present systematic review. Of the 18 reports sought for retrieval, 15 others were excluded. In particular: eight were reviews, five did not involve orthodontic subjects with (at least) one positive reaction to allergy testing, in one it was impossible to extract data from orthodontic subjects, and one was an in vitro study.

Finally, three studies [47,48,49] were included via the manual search process.

In total, 31 studies [1,4,12,22,23,24,25,26,27,28,29,30,31,32,33,34,35,36,37,38,39,40,41,42,43,44,45,46,47,48,49] concerning orthodontic subjects with (at least) a positive reaction to an allergy test before and/or during and/or after orthodontic treatment, retrieved from electronic and manual searches, were included in this systematic review.

**Figure 2:**
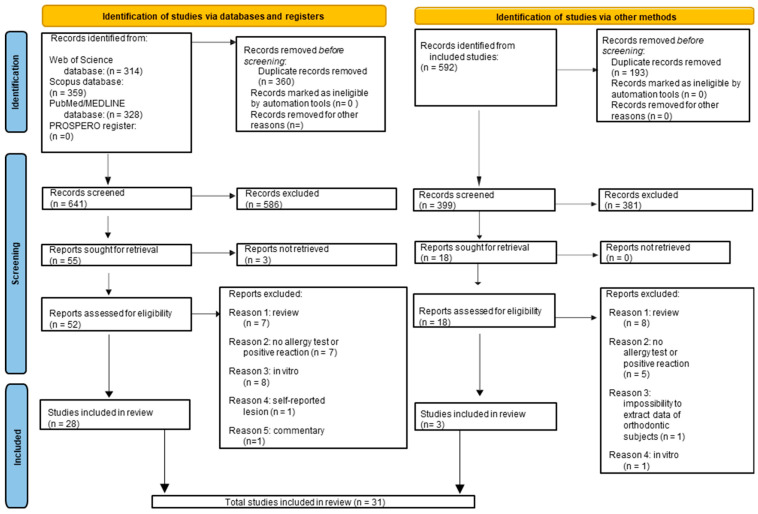
PRISMA 2020 flow chart for the electronic identification of studies retrieved through databases and via other methods.

The flow chart in Figure 2 illustrates the study selection.

### 3.2. Study Characteristics

Of the 31 studies included [1,4,12,22,23,24,25,26,27,28,29,30,31,32,33,34,35,36,37,38,39,40,41,42,43,44,45,47], 14 were case reports [22,24,25,26,27,28,32,34,35,36,39,41,42,44], 6 cross-sectional [12,31,40,45,48,49], 5 prospective [1,23,30,46,47], 3 case-control [29,33,37], 2 RCTs [4,38], and 1 case series [43], involving the same sample, as declared by the study authors.

The data extracted and collected from the studies included in the present systematic review are shown in Table 1; the results of the RCTs were synthesized qualitatively considering the common sample.

### 3.3. Study Population: Orthodontic Subjects

As participants took part in the two currently included RCTs [4,38] and in three cross-sectional studies [45,48,49] and the exclusive extraction of data from orthodontic subjects, the results of a total of 858 orthodontic subjects with at least one positive reaction to allergy test subjects [1,4,12,22,23,24,25,26,27,28,29,30,31,32,33,34,35,36,37,38,39,40,41,42,43,44,45,47,48,49], 114 (13.29%) males and 714 (83.22%) females [1,12,22,23,24,25,26,27,28,29,31,32,33,34,35,36,37,38,39,40,41,42,43,44,45,46,48,49], aged between 9 and 49 years old [1,12,23,29,30,33,37,38,40,43,45,47,48,49], were retrieved. In two studies [30,47], the gender ratio was not provided.

History of allergies was reported in 86 subjects [22,23,25,26,27,28,34,36,37,39,40,44,47], while in one study [30] it was not defined.

Allergy tests were performed before orthodontic treatment on 477 subjects [1,12,23,26,27,28,30,36,37,46,47], while in two studies [34,39] the allergens were not specified.

Other comorbidities were reported in 4 subjects [24,25,26,41]: marginal gingivitis (n = 1) [24], hay fever (n = 1) [25], Von Willebrand’s disease (n = 1) [26], mild asthma (n = 1) [41].

Two subjects were taking pharmacological therapies: antihistamine drugs (n = 1) [25], salbutamol and corticosteroids inhaler (n = 1) [41].

### 3.4. Study Intervention: Orthodontic Treatment and Appliances

All the 858 subjects included in the present systematic review were orthodontic subjects (with any type of orthodontic appliance), as defined by the inclusion criteria.

The type of orthodontic appliances was specified in 743 (86.60%) orthodontic subjects [1,4,12,22,23,24,25,26,27,28,29,30,31,32,33,34,35,36,37,38,39,40,41,42,43,44,45,47], as follows: 656 subjects were treated with FAs [1,12,23,24,25,26,28,29,30,38,39,40,43,45,46,47,48,49], 81 with both FAs and RAs [35,37,41], 3 with intra- and extra-oral RAs [33,36,42], and 3 with CAs [22,27,32]. In the remaining 114 subjects [31,34,44] the type of orthodontic appliances was not available.

The orthodontic material of FAs was described in 18 studies [1,12,23,25,26,28,29,30,38,40,43,45,47,48,49], specifically: stainless steel with Ni or with Ni-Cr; elastic archwires with a high Ni content and stiff archwires with a low Ni content were described in 7 studies [12,23,25,30,34,40,43]; with acrylic resin described in 2 studies [12,43]; with ceramic described in 1 study [23]; with only Ni in 5 studies [1,4,33,45,47]; with Ti described in 4 studies [1,28,37,45]; with Ni-Ti described in 4 studies [23,35,40,45,48,49]; with Ni-Cr described in 3 studies [29,36,43]; with Co [40]; with nickel-free appliances: more than 18% Cr, 0.2–4% Ni, 3.5% Mo [4], and with Mn, Co, Mo, Fe, Cu, and other metals in 3 studies [1,4,26].

The orthodontic material of RA(s) was described in 3 studies [34,36,42], specifically: with stainless steel Ni-Cr in 2 studies [34,36] and with Ni-sulphate in 1 study [42].

The orthodontic material of treatment, both with FAs and RAs, was described in three studies [35,37,41], specifically: with Ti in 1 study [37] and Ni-Ti in 1 study [35]. Orthodontic material was described in 3 studies for CA(s), specifically: with polyurethane described in 1 case [22]; with copolyester described in 1 study [27]; with natural rubber latex, sulfur, zinc oxide, polymeric hindered phenol, and dithiocarbonate derivative in 1 study [32]. The orthodontic material was not reported in four studies [33,41,44,46].

Orthodontic treatment duration was described in six studies [27,28,31,33,35,41] and ranged from 6 weeks to 40 months (mean: 21.5 months).

Orthodontic treatment in which there had been a discontinuation/device substitution was recorded in twelve studies [22,24,25,26,32,34,35,36,39,41,42,43]. In particular, in seven studies [22,25,32,34,39,41,42] the orthodontic treatment was discontinued, in four studies [24,26,35,36] the device was replaced, and in one study [43] both a discontinuation and a device replacement occurred.

### 3.5. Manifestations Potentially Associated with Hypersensitivity Reactions with Oral and/or Extra-Oral Involvement in Orthodontic Subjects: Prevalence and Features

**Figure 3:**
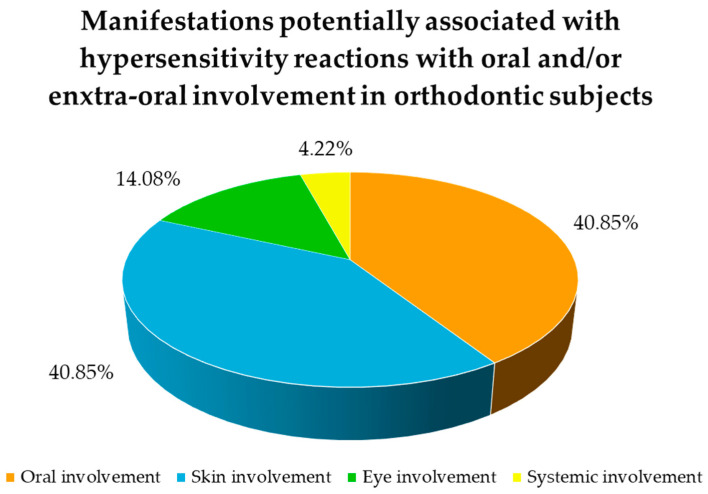
Manifestations potentially associated with hypersensitivity reactions with oral and/or extra-oral involvement (absolute number; percentage in relation to the total number of involvement) in orthodontic subjects.

Oral involvement was described in the 40.85% times of the reported cases [22,24,32,35,41,43,44,45,46,48,49], while the extra-oral involvement skin involvement 40.85% times [22,24,25,26,34,35,36,39,41,42,43,44,45,48,49], eye involvement 14.08% times [25,36,45], and systemic involvement 4.22% times [45,48,49], as shown in Figure 3.

#### 3.5.1. Manifestations Potentially Associated with Hypersensitivity Reactions with Oral Involvement in Orthodontic Subjects: Prevalence and Features

Oral involvement was recorded in 10 studies [22,24,32,35,41,43,44,45,46,48,49].

Burning/stinging was described in 5 studies [22,44,45], gingival hyperplasia in 1 study [24], redness in 1 study [24], soreness in 1 study [32], erythema in 1 study [35], gingival enlargement in 1 study [41], pruritus and/or discomfort of the buccal mucosa in 2 studies [43,46], an itching sensation in 1 study [44], swelling of the tongue in 3 studies [45,48,49], a weakened sense of taste in 3 studies [45], a weakened sense of smell in 3 studies [45], and dysgeusia in 3 studies [45].

The primary oral lesions characterizing the macroscopic features were detailed in 2 studies [32,43], describing vesicles and erosions at lips, buccal mucosa, and gums.

Microscopic features of oral lesions were not reported in any of the included studies.

Definitive oral diagnoses were declared in 4 studies [22,32,43,44], specifically in 2 studies [32,44] as allergic contact stomatitis, in 1 study [22] as angioedema, and as unspecified stomatitis in 1 study [43].

In one study [41], trauma-induced edema following dental extractions, and delayed type IV hypersensitivity reaction to latex [41], were considered as differential diagnoses.

The pharmacological therapies were stated in 4 studies [22,32,41,44]: oral prednisone in 1 study [22], oral antibiotics in 1 study [32], anti-histamine (oral Loratadine 10 mg the night before orthodontic appointments, and 10 mg the day of the procedure in 1 study [41]) and oral and topical corticosteroids in 1 study [44].

The orthodontic treatment was discontinued in two subjects under treatment with CAs.

Lesion resolution/progression was found in six studies [22,24,32,35,41,43] with healing after device removal or substitution, or healing after pharmacological therapy [41,44]. 

#### 3.5.2. Manifestations Potentially Associated with Hypersensitivity Reactions with Extra-Oral Involvement in Orthodontic Subjects: Prevalence and Features

Extra-oral involvement was recorded in 14 studies [22,24,25,26,34,36,39,41,42,43,44,45,48,49].

Skin involvement was described as erythema with/without itchy papules in 5 studies [25,36,39,42,44]; fissurations and scaly itching lesions in 3 studies [34,35,44], edema with/without microvesiculation in 3 studies [36,39,41], crusts were in 3 studies [34,39,41], unspecified rash in 2 studies [24,25], vesicles in 2 studies [25,34], swelling in 4 studies [22,45,48,49], burning/stinging in 2 studies [22,44], redness in 2 studies [22,35], desquamation in 1 study [35], soreness in 1 study [35], and pruritus and dryness in 1 study [43].

Eye involvement was described as redness, itching, and tearing in 1 study [25], conjunctival hyperemia of both eyes in 1 study [36], and watery eyes in 3 studies [45,48,49].

Systemic involvement was recorded in three studies as vertigo [45].

Microscopic features were not reported.

Definitive extra-oral diagnoses were declared in 8 studies [22,24,26,32,34,35,43,44] with: dermatitis in 3 studies [24,34,43], eczema in 3 studies [26,34,43], delayed-type hypersensitivity reactions to latex in 1 study [32], allergic contact dermatitis in 2 studies [35,44], urticaria in 2 studies [22,35], and Ni-allergy in 1 study [35].

Pharmacological therapies were carried out in seven studies [1,22,32,34,41,42,44], with oral prednisone [22], oral antibiotics [32], local medication for eczema [34], N/D topical treatment [1], anti-histamine Loratadine 10 mg orally the night before orthodontic appointments and 10 mg the day of the procedure [41], and oral and topical corticosteroids [44].

The orthodontic treatment was discontinued in 3 subjects under treatment with fixed devices [25,39,41] and in 2 subjects under treatment with mobile devices [34,42], while the device was replaced in 2 subjects under treatment with fixed devices [24,26], in 1 subject in treatment with a mobile device [36], and in 1 subject in treatment with a combination of fixed and mobile devices [35].

Lesion resolution/progression was described in 13 studies [22,24,25,26,32,34,35,36,39,41,42,43,44] with healing after device removal or substitution [22,24,25,26,32,34,35,36,39,42,43] or with healing after pharmacological therapy [41,44].

### 3.6. Frequency of Manifestations Potentially Associated with Hypersensitivity Reactions with Oral and/or Extra-Oral Involvement in Relation to the Orthodontic Appliance

Orthodontic subjects in treatment with FAs manifestation(s) potentially associated with hypersensitivity reactions with oral and/or extra-oral involvement were registered 55 times [24,25,26,39,43,45,46,48,49].

In particular, extra-oral involvement was recorded 31 times (amounting to 56.36% of the total number of oral and/or extra-oral involvement in subjects with FAs); specifically, skin involvement was 19 (34.55%) [24,25,26,39,43,45,48,49], eye involvement 9 (16.36%) [25,45,48,49], and systemic involvement 3 (5.45%) [45,48,49]. Intra-oral involvements in FAs subjects were registered 24 times (43.64%) [24,43,45,48,49].

Orthodontic subjects in treatment with RAs manifestation(s) potentially associated with hypersensitivity reactions with oral and/or extra-oral involvement were registered four times [34,36,42], amounting to 100% of the total number of oral and/or extra-oral involvement in subjects with RAs recorded. Specifically, skin involvement was three (75.00%) [34,36,42], and eye involvement one (25.00%) [36]. No systemic or oral involvement was retrieved in orthodontic subjects with RAs.

Orthodontic subjects in treatment with both FAs and RAs, manifestation(s) potentially associated with hypersensitivity reactions with oral and/or extra-oral involvement were also registered four times [35,41]. In detail, extra-oral involvement was recorded two times (amounting to 50.00% of the total number of oral and/or extra-oral involvement in subjects with both FAs and RAs) with skin involvement [35,41], while no data on eye or systemic involvement were found in the included studies. Intra-oral involvement was registered two times (50.00%) [35,41].

In orthodontic subjects in treatment with CAs, manifestations potentially associated with hypersensitivity reactions with oral and/or extra-oral involvement were registered three times [22,32]. Extra-oral involvement was recorded one time (amounting to 33.33% of the total number of oral and/or extra-oral involvement in subjects with CAs) as one skin involvement (33.33%) [22], while no data on eye or systemic involvement were found in the included studies. Intra-oral involvement was registered two times (66.67%) [22,32].

**Figure 4:**
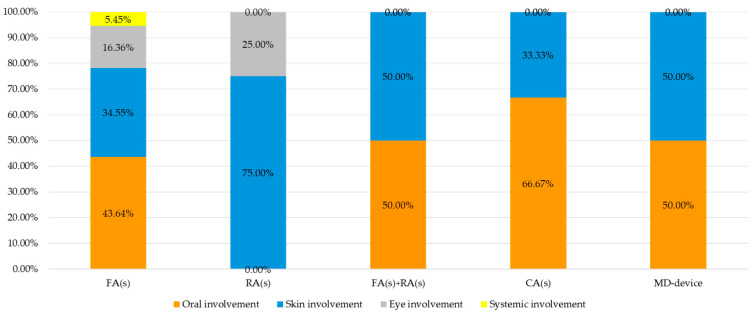
Frequency of manifestation(s) potentially associated with hypersensitivity reactions with oral and/or extra-oral involvement (percentage in relation to the total number of involvements) in relation to the orthodontic appliance.

In one study [44], the manifestations potentially associated with hypersensitivity reactions with one oral and one extra-oral (skin) involvement were also recorded, but the orthodontic appliances were not specified. The frequency of manifestation(s) potentially associated with hypersensitivity reactions with oral and/or extra-oral involvement in relation to the orthodontic appliance is shown in Figure 4.

#### 3.6.1. Manifestations Potentially Associated with Hypersensitivity Reactions with Oral Involvement: Relative Frequency Related to the Orthodontic Treatment Appliance and Characterization

**Figure 5:**
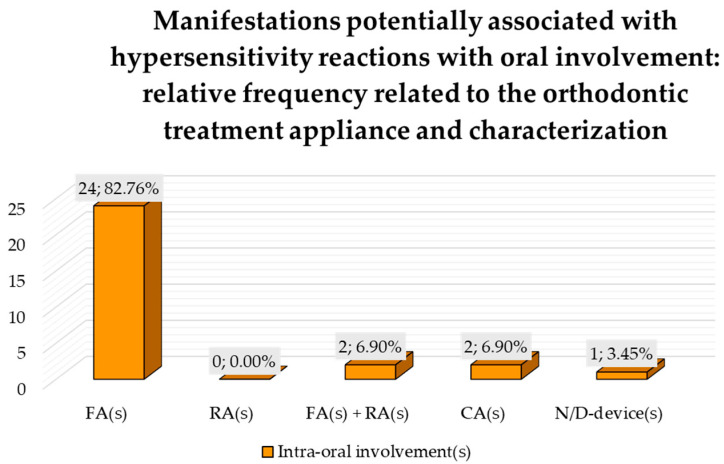
Frequency of manifestations potentially associated with hypersensitivity reactions with oral involvement (absolute number; percentage in relation to the total number of reports) in relation to the orthodontic appliance.

Manifestations potentially associated with hypersensitivity reactions with oral involvement were reported in 82.76% of cases among subjects under treatment with FAs [24,43,45,46,48,49], representing 43.64% of FAs treatment; in 6.90% of reports in patients with both FAs and RAs [35,41], corresponding to 50% of such combined treatments, in 6.90% of reports with CAs [22,32], corresponding to 66.7% of treatment with CAs, and in 3.45% of reports with unspecified orthodontic appliances [44]; no oral involvement was recorded in orthodontic subjects with RAs (Figure 5). The time to onset ranged from 1 day to 4 weeks after device application (mean time: 15 days).

#### 3.6.2. Manifestations Potentially Associated with Hypersensitivity Reactions with Extra-Oral Involvement: Relative Frequency Related to the Orthodontic Treatment Appliance and Characterization

**Figure 6:**
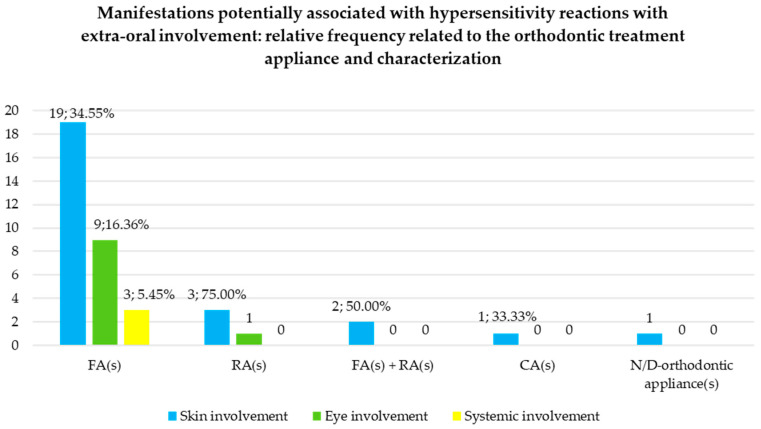
Frequency of manifestations potentially associated with hypersensitivity reactions with extra-oral involvement (absolute number; percentage in relation to the total number of reports) in relation to the orthodontic appliance.

Manifestations potentially associated with hypersensitivity reactions with extra-oral involvement were described 31 times (79.49% of reports) in relation to FAs, 4 times (20.26% of reports) in relation to RAs, 2 times (5.13% of reports) in relation to FAs and RAs, 1 time (2.56% of reports) in relation to CAs, and 1 time (2.56% of reports) in relation to unspecified orthodontic appliances (Figure 6). The time to onset ranged from 1 day to 4 weeks after device application (mean time: 15 days), but it was not possible to define it for the different orthodontic devices or the various affected extra-oral areas.

In particular, skin involvement was described 26 times in total, and specifically 19 times in subjects under treatment with FAs [24,25,26,39,43,45,48,49] amounting to 34.55% of FAs treatment, 3 times with RAs [34,36,42], amounting to 75.00% of RAs treatment, 2 times with both FAs and RAs [35,41], amounting to 50.00% of both FAs and RAs treatment, 1 time with CAs [22], amounting to 33.33% of CAs treatment, and 1 time with unspecified orthodontic appliances [44].

Eye involvement was found 10 times, and specifically 9 times in subjects in treatment with FAs [25,45,48,49], amounting to 16.36% of FAs treatment, and 1 time with RAs [36], amounting to 25.00% of RAs treatment. No eye involvement was recorded in orthodontic subjects with both FAs and RAs, and CAs.

Systemic involvement was recorded three times, and all of them in orthodontic subjects with FAs [45,48,49], amounting to 5.45% of FAs treatment; no systemic involvement was recorded in orthodontic subjects with RAs, both FAs and RAs, and CAs.

### 3.7. Hypersensitivity Status Before, During, and After the Orthodontic Treatment: Tests, Allergens, Timing

History of allergies was reported in 86 orthodontic subjects [22,23,25,26,27,28,34,36,37,39,40,44,46,47,48,49], specifically allergy to nickel in 41 subjects [23,26,27,28,36,37,47], contact hypersensitivity to nickel in 19 subjects [45,48,49], contact hypersensitivity to imitation jewelry in 14 subjects [45,48,49], contact hypersensitivity to metal in 6 subjects [45,48,49], allergy to Penicillin in 2 subjects [22,44], atopy in 2 subjects [34,39], allergy to Amoxicillin in 1 subject [22], allergy to Chromium in 1 subject [28], allergy to eyeshadows in 1 subject [36], and allergy to dust and some kind of mascara in 1 subject [26]. In three subjects, allergy was not specified [40], while in one study [30] it was not defined.

Allergy test(s) performed totalled 1283 [1,4,12,22,23,24,25,26,29,30,31,32,33,34,35,36,37,39,40,41,42,43,44,45,46,47,48,49]: 1257 patch tests, 5 prick tests, 4 oral challenge tests, 8 blood tests, 7 lymphocyte proliferation tests, 1 N/D allergy test to Ni and latex, and one unspecified allergy test [27].

The timing of the allergic test performed was reported in 8 studies [1,23,29,30,45,46,48,49]. Specifically, allergy tests were performed before orthodontic treatment 471 times (469 patch test [1,12,23,26,28,30,36,37,47], one blood and lymphocyte proliferation tests [46], one specific allergy test [27]), during the orthodontic treatment 70 times (66 patch test and 4 blood and lymphocyte proliferation tests [29,46]), and after the orthodontic treatment 768 times (754 patch test [1,4,12,22,23,24,25,29,30,31,32,33,34,35,36,39,40,42,43,44,45,46,47,48,49], 5 prick test [36,43], 4 oral challenge [43], 3 blood test [26,41,46], 2 lymphocyte proliferation test [26,46], and 1 allergy test unspecified [41]).

Several allergens were tested: nickel (Ni), cobalt (Co), chromium (Cr), manganese (Mn), molybdenium (Mo), fragrance mix, gress pollen, nickel-titanium (Ni-Ti), titanium (Ti), thiomersal, silver (Hg), palladium (Pa), rubber bands, Invisalign aligner materials, diaminodiphenylmethane and hexamethylene diisocyanate.

**Figure 7:**
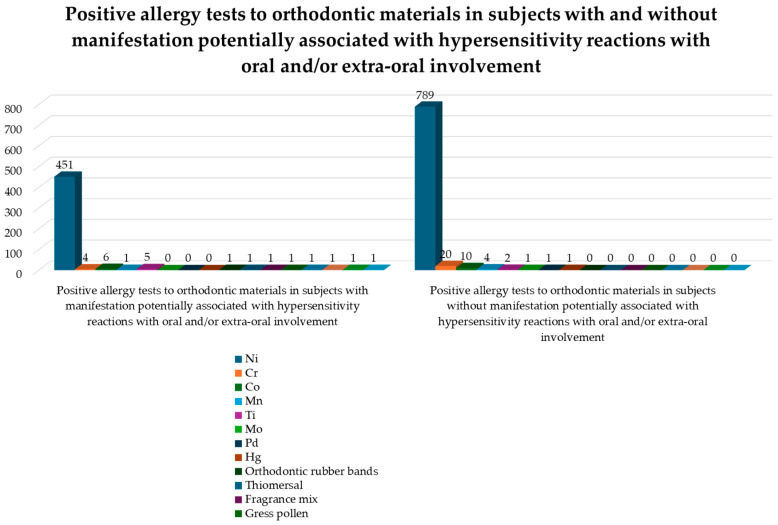
Positive allergy tests to orthodontic materials in subjects with and without manifestation potentially associated with hypersensitivity reactions with oral and/or extra-oral involvement.

Positive allergy tests to orthodontic materials in subjects without manifestations have been reported for: Ni 789 times [1,4,12,23,27,28,29,30,31,33,37,40,46,47], Cr for 20 times [1,28], Co for 10 times [28,40], Mn for 4 times [1], Ti for 2 times [1], Mo for 1 time [1], Pd for 1 time [46], and Hg for 1 time [1] (Figure 7).

Positive allergy tests to orthodontic materials in subjects with manifestations potentially associated with hypersensitivity reactions were reported for: Ni 451 times [12,24,25,28,34,35,36,39,42,43,45,46,48,49], Co 6 times [25,28,34,43], Ti 5 times [45,48,49], Cr for 4 times [28,43], and 1 time for Mn [44], orthodontic rubber bands [32], Thiomersal [35] Ni-sulphate [42], grass pollen [43], Pa [25], Invisalign aligner materials [22], diaminodiphenylmethane and hexamethylene diisocyanate [22] (Figure 7).

### 3.8. Study Quality Assessment

Fifteen studies were judged as critical, five studies as serious, and nine studies as moderate risk of bias through the Risk of Bias Instrument for Non-randomized Studies of Exposures [20], and two studies as high risk of bias through the Revised Cochrane risk-of-bias tool for randomized trials (RoB 2) [21], as illustrated in Tables S1 and S2 (Supplementary File S1), respectively.

## 4. Discussion

The primary aim of the present systematic review was to analyze the prevalence, clinics, and microscopy of manifestations potentially related to hypersensitivity reactions with oral (mucosal lesions, hypo-/dysgeusia +/− hyposmia, dysesthesia) and extra-oral (skin, eye, and systemic) involvement in orthodontic subjects with almost one positive reaction to an allergy test before and/or during and/or after orthodontic treatment, assessing their relative frequency in patients treated with fixed or removable appliances or clear aligners.

The secondary objectives were to comprehensively evaluate reported manifestations according to patients’ age, gender, (ascertained) family and/or medical history of positive reactions to allergy tests and (any) hypersensitivity manifestations prior to orthodontic treatment, comorbidities and associated ongoing therapies, as well as orthodontic appliance, time since initiation and total duration of orthodontic treatment, and need for interruption and/or appliance replacement, to provide clinical recommendations for managing orthodontic treatment in patients with almost one positive reaction to an allergy test before and/or during orthodontic treatment.

Thirty-one studies [1,4,12,22,23,24,25,26,27,28,29,30,31,32,33,34,35,36,37,38,39,40,41,42,43,44,45,46,47,48,49] were included in the present systematic review, and a total of 75 oral and extra-oral involvement (oral involvement 29 times, skin involvement 26 times, eye involvement 10 times, and systemic involvement 3 times) potentially associated with hypersensitivity reactions were registered in the 858 orthodontic subjects with (at least) one positive reaction to an allergy test involved linked with both FAs, Ras, and CAs.

### 4.1. Manifestations Potentially Associated with Hypersensitivity Reactions with Oral and/or Extra-Oral Involvement in Orthodontic Subjects: Prevalence and Features

#### 4.1.1. Manifestations Potentially Associated with Hypersensitivity Reactions with Oral Involvement in Orthodontic Subjects: Prevalence and Features

Oral manifestation(s) potentially associated with hypersensitivity reactions in orthodontic subjects were reported 29 times and were registered predominantly in the female gender (gender ratio M:F = 0:11), while the mean age was 23.7 years.

The female gender is generally more prone to abnormal immune system reactions and immune-inflammatory dysregulation [50], probably due to immune and hormonal factors [51]. The female gender was also most inclined to periodontal, peri-implant, and orofacial reactions, as reported by Feller et al. [52] (gender ratio M:F = 1:2.67). These findings may also be explained considering the continuous contact with cosmetic products, which contain several metals, such as Ti and Ni, in the female gender compared to males [53]. Indeed, the major incidence in females of oral manifestations potentially associated with hypersensitivity reactions to metals in orthodontic subjects or subjects with dental implant-supported prosthesis may be the manifestations of repeated exposures for many years to the allergen/antigen at sub-threshold concentrations [52,53].

Further evidence of the higher risk of developing oral manifestations potentially associated with hypersensitivity reactions among the female gender, was also found in relation to the oral drug adverse reactions, following the anti-SARS-CoV-2 vaccines, as well as anti-influenza vaccines, anti-yellow fever, anti-morbillus-varicella-rubella, and anti-Japanese encephalitis virus [54,55]. In fact, oral manifestations following anti-SARS-CoV-2 vaccines were registered more frequently in females (68.8%) than males (31.2%) [55]. The major incidence of oral manifestations following vaccinations in the female gender was probably associated with the gender differences in adipose tissue distribution, body mass index, and pharmacodynamics [56].

Macroscopically, the lesions consisted of vesicles/bullae and erosions localized to the lips, buccal mucosa and gingiva, as described in two studies [32,43]. The macroscopic features of the lesions appear in some cases to be similar to those found after administration of the anti-SARS-Cov-2 vaccine, where desquamation, hemorrhagic eschars, vesicles and bullae were noted, in addition to white plaques, papules and maculae [55]. Of note, the last oral mucosal lesions, often defined as lichenoid lesions or (contact) reactions [7,57], were presently never described in orthodontic patients, even after administration of the anti-SARS-Cov-2 vaccine in subjects < 18 years of age, as opposed to adults, probably suggesting that oral lichenoid lesions may be more common in adults compared to younger subjects. This observation is even more relevant considering that oral lichenoid lesions are commonly observed near metallic restorations and prosthetic rehabilitations [53].

The microscopic features of oral lesions were not detailed in the studies included. However, they generally included intense vascular proliferation, inflammatory infiltrates characterized by mixed abundant macrophages, chronic inflammation foci, subacute and moderate, granulation tissue, and giant cells [53,58].

A definitive diagnosis included allergic contact stomatitis [32,44], angioedema [22], and an undefined stomatitis [43]. This finding, according to Feller et al. [52], could be related to the salivary flow, which, although it clears specific potentially exogenous allergenic agents from direct contact with epithelial cells and abundant blood supplies, possibly allows the development of hypersensitivity reactions of the oral mucosa due to a dysregulation of oral mucosal immunity.

Pharmacological treatment of hypersensitivity reactions in orthodontic patients was described in four studies [22,32,41,44].

One case [32] of stomatitis was initially misdiagnosed as an infection and treated with antibiotics [31], with lesions resolving within two weeks after discontinuing the elastics.

In another case [22], oral prednisone in a topical solution was administered in a patient with an allergic history, including asthma, although the burning and stinging of the oral mucosa persisted until the aligner was removed, leading to symptom resolution. Indeed, prednisone or antihistamines were deemed effective in some instances (freely available online on https://www.msdmanuals.com/professional/immunology-allergic-disorders/allergic-autoimmune-and-other-hypersensitivity-disorders/overview-of-allergic-and-atopic-disorders, accessed on 31 March 2024), especially in relation to type IV (delayed hypersensitivity) reactions, which involve T cell sensitization following antigen exposure. Upon re-exposure, these T cells activate and cause tissue damage either directly or through the release of cytokines, which in turn activate eosinophils, macrophages, monocytes, neutrophils, or natural killer cells. Histamine release from mast cells and basophils, sensitized by IgE, plays a key role in inflammation and clinical atopy, resulting in symptoms such as erythema, edema, itching, bronchoconstriction, increased gastrointestinal motility, and secretions from nasal, salivary, and bronchial glands. Antihistamines, particularly H1 receptor blockers, are fundamental in treating allergic disorders as they alleviate the symptoms of various atopic and allergic diseases. Their action typically begins within 15–30 min, peaks at around one hour, and lasts for 3–6 h. Additional pharmacological treatments include mast cell stabilizers, corticosteroids for more severe or widespread reactions, nonsteroidal anti-inflammatory drugs (NSAIDs) to alleviate conjunctival injection and itching, leukotriene inhibitors for mild persistent asthma, seasonal allergic rhinitis, and urticaria, and anti-IgE antibodies and immunotherapy in selected cases.

In additional cases, hypersensitivity reactions were managed by replacing the causative devices with alternatives such as acrylic resins, ceramic brackets, or Ni-free devices. This replacement led to symptom resolution and no new lesions during follow-up.

#### 4.1.2. Manifestations Potentially Associated with Hypersensitivity Reactions with Extra-Oral Involvement in Orthodontic Subjects: Prevalence and Features

Skin involvement included erythema, fissuration, edema, eczema, unspecified rash and dermatitis [25,36,39,42,44], clinical signs consistent with type IV hypersensitivity reaction, as noted by Dispensa M. et al. [59]; both contact dermatitis and atopic conditions such as asthma or allergic rhinitis were also assessed. The most common extra-oral cutaneous manifestations were localized to the face, particularly in the perioral area, likely due to increased sensitization of the skin in this area and on the lips from personal care products or the structure of certain devices, such as face masks with chin supports worn for extended periods. Skin involvement was also noted in the periorbital area, eyelids, fingers and neck, probably due to cosmetics, and creams that may have sensitized the area [60].

The microscopic features of extra-oral lesions were not detailed.

Eye involvement manifested as redness, itching, tearing, conjunctival hyperemia of both eyes and watery eyes [25,36,45,48,49]. In fact, the eye is a common site of allergic reaction manifestations, often presenting as allergic conjunctivitis, a condition referring to a cluster of disorders affecting the eyelids, conjunctiva and/or cornea [61]. The ocular involvement could be linked to the common mechanism underlying allergic mechanisms, related to T-cell-mediated responses and specific cytokines and chemokines [61,62,63].

Systemic involvement was recorded in three studies [45,48,49], also including vertigo as a systemic manifestation. Vertigo episodes due to allergic reactions might be possible but are often misdiagnosed. Indeed, in Wu et al.’s study [64], a subject with frequent episodes of vertigo was initially misdiagnosed with Ménière’s disease, and his symptoms improved after he tested positive for allergy and received appropriate treatment [64].

Differential diagnosis was reported in one study [41], including trauma-induced edema after dental extractions, delayed type IV hypersensitivity reaction to latex, and type IV cell-mediated delayed hypersensitivity reaction to nickel. The importance of differential diagnosis cannot be overstated, as the clinical manifestations of IgE-mediated reactions can range from itching, erythema, and localized urticaria to generalized urticaria, angioedema, rhinitis, conjunctivitis, bronchospasm, and anaphylactic shock. These symptoms and clinical signs may arise due to a latex allergy [65] or, as noted in this systematic review, hypersensitivity reactions to other materials. Atopy is considered a risk factor for latex sensitization. In the general pediatric population, latex sensitization occurs in approximately 1%, but this prevalence increases to 3–5% among atopic children, with about half exhibiting clinical manifestations [66]. The diagnosis of a latex allergy relies on the subject’s medical history and is supplemented by in vivo research (e.g., skin and provocation tests) or in vitro studies (such as specific IgE determination) [67].

Pharmacological therapies for extra-oral involvement included oral prednisone, local treatments for eczema, antihistamine loratadine, and oral and topical corticosteroids [22,34,41,42,44]. The approaches described in the reviewed articles align with standard allergy management practices, with treatment tailored to the affected area by the manifestations; for example, topical steroids are used to reduce inflammation in cases of dermatitis [68]. The use of antihistamines in allergic individuals is also crucial (freely available online on https://www.msdmanuals.com/professional/immunology-allergic-disorders/allergic-autoimmune-and-other-hypersensitivity-disorders/overview-of-allergic-and-atopic-disorders, available on 31 March 2024). However, the primary recommendation from several studies is to avoid known allergens or, when possible, remove the causative factor of the manifestations [69]. This review corroborates these findings, as various pharmacological treatments were employed to manage the manifestations, but resolution typically occurred only after the allergen was removed through replacement or removal of the orthodontic device.

The resolution and progression of lesions were described in 12 studies reporting extra-oral involvement [22,24,25,26,34,35,36,39,41,42,43,44], with healing observed after device removal or substitution [21,23,24,25,33,34,35,39,42,43] or after pharmacological therapy [41,44].

### 4.2. The Frequency of Manifestations Potentially Associated with Hypersensitivity Reactions with Oral and/or Extra-Oral Involvement in Relation to the Orthodontic Appliance

All orthodontic devices used in this review have been associated with extra-oral and intra-oral manifestations potentially linked to hypersensitivity reactions, though with varying incidences. The incidence of extra-oral hypersensitivity was 56.36% for fixed devices, 100% for mobile devices (including extraoral face bows and facemasks), 50.00% for fixed plus mobile devices, 33.33% for aligners, and 50.00% for MD-devices. The incidence of intra-oral hypersensitivity was 43.64% for fixed devices, 0% for mobile devices, 25.00% for fixed plus mobile devices, 66.77% for aligners, and 50.00% for MD-devices.

Oral involvement was reported more frequently with FAs [24,45], followed by combined devices FAs + RAs [35,41], CAs [22,32], and MD-devices [44].

Extra-oral involvement was reported mainly with FAs (79.49%), less with RAs (20.3%), combined fixed plus removable appliances (FAs + RAs, 5.1%), CAs (2.56%), and MD-devices (2.56%).

Skin manifestation predominated, especially with FAs (34.55%) and RAs (75.00%), while eye involvement was less common and systemic manifestations rare, occurring in FAs (5.45%).

The prevalence of extra-oral and intra-oral involvement in subjects undergoing treatment with fixed appliances compared to those using mobile appliances and/or aligners could be attributed to greater daily exposure to the metals used in fixed orthodontics, potentially sensitizing the patient. Additionally, the majority of orthodontic patients examined were undergoing treatment with fixed appliances, which may have influenced the results [67,70].

The greater prevalence of skin involvement compared to ocular or systemic involvement is notable. This could be due to the close proximity of the devices to the skin in the perioral and labial areas, aligning with the typical progression of allergic reactions, which often start with skin manifestations such as rashes and urticaria before involving other body structures [67,70].

#### 4.2.1. Orthodontic Treatment Appliance(s)

All 858 subjects included in the present systematic review were orthodontic patients treated with various appliances. The specific device was reported in 743 cases (83.6%) [1,4,12,22,23,24,25,26,27,28,29,30,31,32,33,34,35,36,37,38,39,40,41,42,43,44,45,47], as follows: 656 subjects were treated with FAs [1,12,23,24,25,26,28,29,30,38,39,40,43,45,46,47,48,49], 81 with both FAs and RAs [35,37,41], 3 with intra- and extra-oral RAs [33,36,42], and 3 with CAs [22,27,32]. In the remaining 114 subjects [31,34,44] the type of orthodontic appliances was not available.

Hypersensitivity reactions in fixed orthodontics may arise from metals such as nickel, chromium, titanium, and molybdenum, commonly found in wires, bands, and brackets [71]. Orthodontic bands are also typically made of stainless steel, which may cause local tissue reactions [72,73] depending on the concentration and chemical state of the metal [74,75]. Brackets are usually composed of stainless steel, ceramic, or plastic, but hypersensitivity reactions are more often related to stainless steel due to the nickel and chromium presence in the alloy [74,75].

Moreover, numerous scientific studies [76,77] have described the corrosive action of various beverages and foods on metals used in orthodontic treatments, altering enamel features and affecting the mechanical and physical properties of orthodontic wires, thereby decreasing the rate of orthodontic movement. Recent in vitro studies [78,79] have highlighted that the corrosivity of these metals may lead to the release of particles into the bloodstream, which can activate the immune system and cause hypersensitivity reactions. It was also observed that titanium alloys have a higher corrosion resistance than stainless steel, and the release of ions could depend on changes in pH and the composition of the wire, but was not directly proportional to the metal content in the wire.

The release of particles into the bloodstream is not limited to metal orthodontic devices but should also be considered for aligners. A recent study from 2023 [80] pointed out that even the use of high-fluorine mouthwashes involves the release of metal ions. The release of ions in Ni-Ti wires at high concentrations changes the microstructure, reducing the effectiveness of the wire’s properties, and also causes a nickel ion relapse that can be toxic. Previous or concurrent exposures to metals, which were not evaluated in the present study, may be the cause of hypersensitivity reactions or could act as an adjuvant factor during orthodontic treatment.

Plastic brackets, originally made of acrylic, have recently been replaced with polycarbonate or polyurethane, and reinforced polycarbonate brackets have been introduced with ceramic or fiberglass fillers and/or metal inserts. These advanced brackets are gaining popularity as a solution to the stiffness and strength limitations of conventional polycarbonate brackets [81]. These polymers, including polyurethane and polycarbonate, are also used in orthodontic aligners.

It has been observed that these materials can cause hypersensitivity reactions due to their degradation from temperature fluctuations, pH changes, mechanical wear, and enzymatic activity from bacteria or saliva [82,83,84,85], which can lead to the leaching of bisphenol-A (BPA). BPA is known to have estrogenic and cytotoxic effects and may be associated with an increased risk of prostate and breast cancer [86,87]. However, the literature remains inconclusive, as some in vitro studies suggest that there is no significant estrogenic or cytotoxic potential. It should be noted, though, that in vitro conditions cannot perfectly simulate the unpredictable stresses of chewing, as pH/temperature variations [88].

In the context of mobile orthodontics, most skin reactions detected in the perioral area or facial region may result from direct and prolonged contact with removable extraoral components, such as a face mask. These devices often share components with fixed appliances, which may potentially present similar hypersensitivity reactions.

Alongside the steel components, hypersensitivity reactions may stem from the resin components, especially methyl methacrylate and other additives. To minimize these reactions, some authors advised using proper hot-cured acrylic resins [89].

In the oral cavity, chewing and friction induced by the assumption of acidic beverages and enzymatic activities can lead to increased abrasion and friction on the aligner, resulting in more particle release. Exposure to the plastic in Invisalign aligners caused variation in viability, membrane permeability, and epithelial cell adhesion in saline solution environments, impairing epithelial integrity [90]. This impairment was found to be responsible for the formation of haptens, which can potentially lead to allergies to isocyanates, either systemic or localized to the gingiva. This type of reaction was evident in one case in this review [22], where the subject treated with clear aligners exhibited a hypersensitivity reaction to hexamethylene diisocyanate.

Furthermore, the serial application of aligners at longer intervals is recommended. According to the study, renewing aligners every two weeks could repeatedly expose subjects to new cycles of abrasion, potentially contributing cumulatively to tissue damage [88].

#### 4.2.2. Orthodontic Treatment Duration and Times to Onset

Orthodontic treatment duration ranged from 6 weeks to 40 months (mean: 21.5 months) [27,28,31,33,35,41]. This finding aligns with the average duration of comprehensive orthodontic treatment, generally reported as less than two years [91].

Two included studies [35,41] reported both skin and oral involvement, with treatment durations of 24 [41] and 36 months [35], respectively. Despite the longer overall duration in these cases, hypersensitivity manifestations occurred very early, within four days [35] and one day [41] after the start of orthodontic treatment. Similarly, in the studies reporting oral involvement, onset occurred within the first month, with an average onset time of 15 days [22,24,32,35,41,43,44,45].

These findings suggest that the total duration of orthodontic treatment might not be directly linked to an increased risk of oral and/or extra-oral involvements, which occur independently of the orthodontic treatment duration in the first month of treatment.

The onset of lesions was after about 15 days from the application of the (fixed/removable) orthodontic appliances or clear aligner(s), ranging from 1 day to 4 weeks after device application. This figure is inconsistent with the onset time when hypersensitivity reactions generally occur. In fact, hypersensitivity reactions ranging from type I to type III (according to the classification of Coombs and Gell) manifest within 24 h after exposure to the allergen, while type IV hypersensitivity reactions appear between 12 h and 72 h after exposure to the allergen [92]. The delay observed in the present review may be due to sensitization processes developing progressively over repeated exposures to the antigen/allergen during the initial weeks or months of treatment.

Indeed, several in vitro studies [93,94,95] have shown that metal ion release from orthodontic archwires, particularly Ni, was higher during the first few days post-placement, before reaching a steady state [93,94,95].

Similarly, a recent systematic review with meta-analysis confirmed that the salivary concentrations of nickel and chromium peak within hours to a few days after the placement of bands, brackets, or archwires, then gradually decline to below baseline levels after 2–3 months [96].

It is therefore plausible to hypothesize that sensitization to orthodontic materials occurs during the first few days following the placement of the orthodontic appliance or one of its components, during which the highest levels of metal ion release are reached, exceeding those recorded before treatment, which would explain why the onset of manifestations potentially associated with hypersensitivity reactions was always recorded within the first month in the present systematic review.

#### 4.2.3. Orthodontic Treatment Discontinuation/Appliance Substitution

Several studies reported either orthodontic treatment discontinuation or device substitution [22,24,25,26,32,34,35,36,39,41,42,43]. In most cases, lesions resolved after device removal or substitution, with no recurrence during follow-up. However, these clinical decisions often prevented the full completion of the orthodontic treatment plan for malocclusion correction.

The consistent resolution of both systemic and oral lesions following the treatment discontinuation or appliance substitution, and the absence of new lesions during follow-up in all patients, suggests a potential causal link between hypersensitivity reactions and the orthodontic devices. Nonetheless, this relationship cannot be definitively confirmed, as none of the studies accounted for confounding factors such as dietary or lifestyle exposures. For instance, Ni, a common allergen implicated in hypersensitivity, is widely present in many commonly consumed foods such as tomatoes, tuna, cocoa, certain crustaceans, most mollusks, whole grains, nuts, and cauliflower [97]. A study focusing on the Danish population [97] highlighted that the high nickel content in foods typically included in the average diet can cause systemic reactions, such as hand eczema.

Additionally, tattoos represent another potential source of sensitizing metals, since according to Schreiver et al. [98], both tattoo inks and needle tips can contain nickel and chromium. The wear of these needles may cause further nickel exposure, which could potentially relate to the formation of tattoo allergies and systemic sensitization, though this impact is yet to be fully assessed.

### 4.3. Hypersensitivity Status, Tests, and Timing in Orthodontic Patients with and Without Manifestations Potentially Due to Hypersensitivity Reactions

#### 4.3.1. Hypersensitivity Status

Subjects with a history of hypersensitivity numbered 86 [22,23,25,26,27,28,34,36,37,39,40,44,47], while in one study [30] was not defined.

In some cases, hypersensitivity was self-reported by patients without a diagnostic match or allergen identification, highlighting a gap in diagnostic confirmation. Vaillant et al. emphasized the importance of recognizing atopy in patients before starting treatment, as it may influence the patient’s response to allergens [99].

The higher rate of positive tests among females compared to males (M = 114; F = 714) could be due to an increased exposure to sensitizing substances like metals in cosmetics and jewelry. This hypothesis is supported by numerous studies that confirm the presence of metals, such as nickel, chromium, and cobalt, in various body care products. Furthermore, exposure to these metals from non-dental sources can prime the immune system, leading to heightened reactivity upon exposure to orthodontic materials [100,101,102,103].

The incidence of hypersensitivity reactions can be higher with certain materials and types of appliances. Immediate management involves the administration of antihistamines or corticosteroids, with epinephrine in severe cases [12]. Janson et al. [29] found that despite the absence of clinical symptoms, 66 patch tests were positive during treatment, highlighting the need for regular monitoring [29].

The different allergens tested were Ni, rubber bands, Co, Cr, Mn, Mo, fragrance mix, gress pollen, Ni-Ti, Ti, Thiomersal, Hg, Pa, Invisalign aligner materials, diaminodiphenylmethane and hexamethylene diisocyanate.

The most frequently reported allergen was Ni (1240 times), followed by Cr (24), Co (13), and Ti (7). Others, such as Mn, orthodontic rubber bands, Thiomersal, and Invisalign aligner materials, were reported once in each case. 

The high number of positive tests to Ni can be attributed to its widespread presence in many everyday items, including foods, jewelry, personal care products, and even water from nickel-contaminated pipes [60,97,104,105].

Titanium is a material widely used in the manufacture of medical and dental devices due to its biocompatibility [106]. In the medical field, for example, it is used for the manufacture of pacemakers, stents, and endoprostheses, while in the dental field it has been used in orthodontic appliances, dental implant-supported prostheses, and crowns [106]. In this review, positive allergy tests to Ti were recorded five times, with three cases showing skin involvement (swelling of the face). Accordingly, even recent evidence in dental implantology suggests that hypersensitivity to Ti and its alloys may occur, should not be underestimated, and may cause dental implant failures [53,106]. In fact, it was hypothesized that hypersensitivity reactions to Ti could alter the bone turnover, trigger the local inflammatory host response and so mimic peri-implantitis disease in the early stage of implant placement [53]. Other reported oral and extra-oral manifestations potentially related to hypersensitivity to dental-implant Ti and its alloys were facial eczema, orofacial erythema, lip swelling, atopic dermatitis, urticaria, and several described manifestations of gingival overgrowth [53,106,107]. In addition, similar to the macroscopic features recorded regarding dental implants, in subjects with hypersensitivity to Ti and its alloys, no oral lichenoid lesions, or oral lichenoid contact lesions, frequently topographically linked to the putative causative material, were found in this systematic review, either in relation to Ti or with other materials [53,106,107].

#### 4.3.2. Hypersensitivity Tests and Timing

Several types of tests can be performed to detect allergies, according to the clinical context and patient-specific factors. Patch tests are the preferred method for diagnosing delayed hypersensitivity reactions, while prick tests and blood tests are more suitable for immediate reactions [100,101,102,103]. Histopathological examination is useful for a definitive diagnosis in ambiguous cases. Combining these methods allows for the comprehensive evaluation and effective management of hypersensitivity reactions to orthodontic materials [100,101,102,103].

In the reviewed articles, patch tests were the most commonly used, followed by prick tests, blood tests, lymphocyte proliferation tests, and oral challenge tests. Such results are in accord with the evidence supporting that the gold standard for diagnosing contact hypersensitivity reactions is considered the patch test, which is also very useful for diagnosing reactions caused by contact with orthodontic materials [11]. Indeed, patch tests are especially effective for diagnosing T-cell-mediated, delayed-type hypersensitivity reactions, typically occurring 48–72 h after allergen exposure. This method helps identify specific allergens that cause allergic contact dermatitis [2]. The customization of patch tests using dental materials, including orthodontic brackets, elastics, and wires, can directly assess the sensitivity to the materials themselves [59]. This test is particularly valuable due to its sensitivity and specificity, between 70% and 80%, and involves applying a small load of putative allergens to the skin, commonly on the back. After covering these using an adhesive patch, which should cover the skin for 48 h, they are examined for eventual reactions at 48 h and again at 72–96 h. A positive test result is manifested by the appearance of erythema, edema, or vesicles at the site of the allergen application. The European standard battery is the most commonly used series of allergens [18].

Prick tests evaluate IgE-mediated allergic reactions, caused, for example, by latex or certain metals, and consist of placing a drop of the allergen on the skin and pricking through the drop into the epidermis [108]. A positive finding produces a wheal and flare reaction within 15–20 min, when immediate hypersensitivity is suspected [108].

Blood tests, such as specific IgE antibody tests, are employed to evaluate type I hypersensitivity reactions [3,38], measuring IgE antibody load specific to different allergens in the blood, with the ImmunoCAP or Radioallergosorbent Test being particularly effective. Blood tests are valuable for subjects who cannot perform skin testing due to severe manifestations of dermatitis or any other contraindications [3]. They provide crucial information about the patient’s allergic status and are useful in cases where skin tests are not feasible [2,4,67].

The examination of histopathologically affected oral tissue biopsy specimens is particularly useful to confirm delayed-type hypersensitivity reactions by identifying characteristic cellular variations, in particular in ambiguous cases in which both clinical and patch test findings are not conclusive [59]. However, this was not reported in the included studies.

The timing of the allergic test encompassed three main phases in the included studies: before, during, and after orthodontic treatment [1,23,29,30,45,46,48,49]. Before treatment, 471 tests were performed, mainly patch [1,12,23,26,28,30,36,37,47], along with a few blood and lymphocyte proliferation tests [46], and other allergy tests [27]. Pre-treatment testing is crucial in subjects with a well-established history of allergies, enabling the identification of potential allergens and the selection of hypoallergenic orthodontic materials, thereby preventing the hypersensitivity reaction development [100].

During the orthodontic treatment, 70 tests were reported, mostly patch tests and blood and lymphocyte proliferation tests [29,46]). Testing during this stage is essential when symptoms such as erythema, swelling, or ulceration occur, enabling the timely intervention and management of adverse reactions [101]. Post-treatment testing is crucial for subjects who develop delayed hypersensitivity reactions following the removal of orthodontic appliances, guiding future dental treatments by ensuring materials that caused reactions are avoided in further therapy [102,103].

After the orthodontic treatment, 768 tests were conducted, predominantly patch tests [1,4,12,22,23,24,25,29,30,31,32,33,34,35,36,39,40,42,43,44,45,46,47,48,49], alongside prick tests [36,43], oral challenge tests [43], blood tests [26,41,46], lymphocyte proliferation tests [26,46], and other allergy tests [41]. Post-treatment testing is beneficial for identifying delayed hypersensitivity reactions that may not have manifested during treatment, guiding future dental care by avoiding triggering materials, monitoring patient health, and supporting patient education on allergen avoidance.

It is worth noting that the included studies in the present systematic review ranged within a considerable time frame, during which significant advancements have occurred in both orthodontic materials and allergy testing techniques. The potential influence of this temporal heterogeneity should be considered when interpreting the reported data. Indeed, the evolution of manufacturing technologies, the introduction of novel biocompatible materials, and improvements in diagnostic protocols may have influenced the type and frequency of hypersensitivity manifestations over time.

### 4.4. Clinical Recommendations for Managing Orthodontic Treatment in Patients with Almost One Positive Reaction to an Allergy Test Before and/or During Orthodontic Treatment

Before undergoing orthodontic treatment, comprehensive pre-treatment evaluations are essential to identify potential allergens. These evaluations include detailed subject histories and related allergy testing. Patch or specific IgE tests assist in the choice of hypoallergenic materials, like titanium/ceramic brackets, for subjects with a documented metal allergy history [3]. Accordingly, several studies included in the present systematic review reported avoiding traditional devices in nickel-sensitive subjects, opting instead for ceramic brackets, aligners, stainless steel, or low-nickel materials [23,37].

Patient counseling on the potential risks, different treatment options for individuals positive to the allergy test, and the importance of maintaining good oral hygiene is critical for prevention and management [11].

During orthodontic treatment, regular follow-ups to monitor any signs and symptoms and early intervention are crucial to prevent severe hypersensitivity reactions [12]. In the study by Janson et al. [29], allergy tests conducted during orthodontic treatment revealed 66 positive patch tests despite the absence of oral and extra-oral manifestations. The immediate management of reactions includes administering antihistamines or corticosteroids, as noted in studies by Awosika et al., Shargill et al., and Velàsquez et al. [22,41,44], with epinephrine reserved for severe cases [7]. Topical corticosteroids are effective for managing mild reactions, while systemic corticosteroids or antihistamines are necessary for severe reactions [3]. It is also essential to remove the offending material or appliance to prevent additional exposure [5]. In the studies examined, most of the subjects have in fact undergone, following the appearance of oral and/or extra-oral lesions, the replacement of the device [22,24,32,41], while in other cases, the latter was removed before the end of treatment [22,25,32,34,39,41,42].

Frequent and regular follow-ups should be scheduled to monitor any recurring signs of hypersensitivity reactions to ensure patient safety and allow adjustments to minimize discomfort and treatment disruption [6,8].

To minimize the risk of hypersensitivity reactions in orthodontic patients undergoing treatment with fixed appliances, selecting orthodontic materials that are nickel-free or characterized by low nickel content may be preferred—for example, titanium or ceramic brackets, along with titanium-molybdenum alloy (TMA) wires—especially for patients with a documented sensitivity to nickel. For mild allergic manifestations, the use of topical corticosteroids is indicated to alleviate local symptoms of inflammation and discomfort, while more severe reactions may require the administration of systemic corticosteroids or antihistamines. Furthermore, patient education on recognizing the early signs of hypersensitivity facilitates prompt reporting and timely intervention. Finally, regular follow-up appointments are critical to monitor treatment responses and to perform any necessary adjustments to the orthodontic appliances [109].

In orthodontic subjects with removable appliances or clear aligners, replacing conventional materials with hypoallergenic options like silicone-based components may be advisable, accompanied by the same principles of symptom management, oral hygiene, and follow-up [109].

Additionally, the management of hypersensitivity should consider the patient’s overall oral health condition. Indeed, the presence of periodontal disease requires particular caution when applying orthodontic forces [110]. A compromised periodontium may not tolerate standard biomechanical loads, and hypersensitivity-related inflammation may further exacerbate periodontal breakdown. Recent evidence [110] highlighted that orthodontic forces applied to a diseased periodontium increase mechanical stress on periodontal tissues, highlighting the importance of both material biocompatibility and force modulation in susceptible individuals.

### 4.5. Limitations, Points of Strength, and Future Perspectives

The limitations of the present systematic review pertain to the general lack or inaccuracy of data reported, preventing meta-analysis. Additionally, in subjects with both extra-oral and intra-oral involvement, the oral manifestations of hypersensitivity reactions were often documented by healthcare providers not specialized in oral pathology, using varied terminologies, complicating the identification and classification of primary oral lesions. Moreover, the included studies did not register data on the microscopic features of the manifestations, likely due to the clinical preference for non-invasive management, as well as the frequent spontaneous resolution of symptoms following the discontinuation of orthodontic treatment or drug therapies, precluding a histological confirmation of clinical diagnoses and detailed insights into the potential hypersensitivity reactions. Furthermore, data on non-orthodontic-related allergens, such as patients’ lifestyles, including nutrition, cosmetics, tattoos, and the use of carbonated drinks or mouthwashes, were not available, thus precluding a definitive attribution of recorded oral and extra-oral involvements to orthodontic metals, despite an observed resolution of symptoms in most subjects following appliance removal or replacement. Another limitation is represented by the heterogeneity of the study designs included in the present systematic review, which ranged from case reports and case series to cross-sectional studies and randomized controlled trials. The variability in study designs may have influenced the quality and consistency of the reported data, as well as the overall interpretation and generalizability of the reported findings. Moreover, the included studies spanned a wide temporal range, from 1993 to 2024. Over such a prolonged period, relevant changes may have occurred concerning orthodontic materials and manufacturing processes, as well as diagnostic approaches for hypersensitivity reactions, which could have potentially led to an underestimation or overestimation of findings.

However, the present systematic review may be the first to investigate the prevalence, clinical features, and microscopy of manifestations potentially related to hypersensitivity reactions with oral and extra-oral involvement in orthodontic subjects with almost one positive reaction to an allergy test before and/or during and/or after orthodontic treatment, in order to assess their relative frequency in patients treated with fixed (FA) or removable (RA) appliances or clear aligners (CAs), and to evaluate them according to patients’ age, gender, (ascertained) family and/or medical history of positive reactions to allergy tests and (any) hypersensitivity manifestations prior to orthodontic treatment, comorbidities and associated ongoing therapies, as well as orthodontic appliance, time since initiation and total duration of orthodontic treatment, and need for interruption and/or appliance replacement, to provide clinical recommendations for managing orthodontic treatment in patients with almost one positive reaction to an allergy test before and/or during and/or after orthodontic treatment.

Further studies are needed to elucidate the involvement of intra-oral and extra-oral primary lesions potentially associated with hypersensitivity reactions in orthodontic subjects and to assess the role of pre-treatment hypersensitivity status, comorbidities, orthodontic treatment duration, non-orthodontic-related potential allergens (nutrition, cosmetics, tattoos, and the use of carbonated drinks or mouthwashes) as risk factors for sensitization in this population.

## 5. Conclusions

The systematic review included 31 studies, involving 858 subjects (114 males, 714 females, aged 9–49 years), 86 of whom had a history of allergy. Among these, 743 subjects used recorded appliances, predominantly fixed appliances, with a mean treatment duration of 21.5 months.

Among 75 reports, 29 (38.67%) described oral involvement such as burning, gingival hyperplasia, erythema, and vesicles, while 46 (61.33%) had skin, eye, and systemic involvement, including erythema, papules, conjunctival hyperemia, and vertigo. Manifestations potentially related to hypersensitivity reactions were most frequent with fixed appliances, followed by removable appliances and clear aligners. Nickel emerged as the most frequently identified allergen, followed by cobalt, titanium, and chromium.

These findings emphasize the importance of a detailed pre-treatment assessment, including allergy history and testing, especially in atopic or allergic individuals, for identifying potential allergens and selecting hypoallergenic materials in sensitized patients. During treatment, regular monitoring and prompt intervention is essential. Removing the allergenic material is essential to prevent further exposure. Regular follow-up is crucial to detect recurrent reactions early. Clinicians should be prepared to adapt materials and treatment strategies to ensure safety and therapeutic continuity.

## Figures and Tables

**Table 1:** Data collected and extracted from the included studies. Source: first author, year, journal, study type and quality, funding. Population: sample size, age range and mean, gender ratio, history of allergies or hypersensitivity (any), previous allergy test, other comorbidities and ongoing pharmacological therapies, piercing. Intervention: orthodontic treatment appliance, type of brackets, type of archwire, orthodontic material, orthodontic treatment duration, orthodontic treatment discontinuation/appliance replacement. Manifestation(s) potentially associated with hypersensitivity reactions with extra-oral involvement: skin involvement, skin location, eye involvement, systemic involvement. Manifestation(s) potentially associated with hypersensitivity reactions with oral involvement: oral mucosal lesions, hypo-/dys-geusia +/− hyposmia, dysestesia; oral macroscopic features, number, distribution, location, microscopic features, time to onset. Diagnosis, therapy, and progression of the manifestation(s) potentially associated with hypersensitivity reactions: allergy test(s), timing of the allergic test performed, evidence of orthodontic material allergy, differential diagnosis, definitive oral and extra-oral diagnosis, pharmacological therapy, treatment, resolution/progression.

Study	Population	Intervention	Manifestation(s) Potentially Associated with Hypersensitivity Reactions with Extra-oral Involvement	Manifestation(s) Potentially Associated with Hypersensitivity Reactions with Oral Involvement	Diagnosis, Therapy and Progression of the Manifestation(s) Potentially Associated with Hypersensitivity Reactions
Awosika, O. 2017 Dermatitis. Case-report [22] Critical No Funding	**Sample:** n. 1 **Mean Age/Range:** 23 y.o. **Gender ratio:** 1F **History of allergies or hypersensitivity:** Allergy to penicillin and Amoxicillin; family history of asthma. **Previous allergy test:** MD **Other comorbidities**: MD **Ongoing pharmacological** **therapies:** MD **Piercing:** MD	**Orthodontic Treatment Appliance:** CAs (Align Technology, Santa Clara, CA). **Type of brackets:** MD **Type of archwire:** MD **Orthodontic material:** polyurethane **Duration:** MD **Discontinuation/Appliance replacement:** removal of aligners	**Skin involvement:** Swelling Redness Burning/stinging **Skin location:** face periorbital extremities flanks lips **Eye involvement:** MD **Systemic involvement:** MD	**Oral mucosal lesions, hypo-/dys-geusia +/− hyposmia, dysestesia:** Burning/stinging **Oral macroscopic features:** MD **Number:** MD **Distribution**: MD **Location:** oral mucosa **Oral microscopic features:** MD **Time to onset:** 2 days after application of aligners.	**Allergy test(s):** patch test **Timing of the allergic test performed:** MD **Evidence of allergy to orthodontic materials:** Strong positive reaction to Invisalign aligner materials: n.1 PS (1F) Ambiguous reactions to formaldehyde, copper sulfate, cobalt, hexamethylene diisocyanate and diaminodiphenylmethane: n.1 PS (1F) **DD**: MD **Definitive oral diagnosis:** Angioedema **Definitive extra-oral diagnosis:** Urticaria **Pharmacological Therapy**: Oral prednisone **Resolution/Progression:** healing after aligners removal
Bass, J.K. 1993 Am J Orthodont Dentofac Orthop Prospective study [23] Critical No Funding	**Sample:** n.7 **Mean Age/Range:** 20.3 y.o.; 12–48 y.o. **Gender ratio:** 1M/6F **History of allergies or hypersensitivity:** n.5 PS to Ni (0M/5F) **Previous allergy test:** patch test **Other comorbidities**: MD **Ongoing pharmacological** **therapies:** MD **Piercing:** MD	**Orthodontic Treatment Appliance:** FA **Type of brackets:** MD **Type of archwire:** MD **Orthodontic material:** stainless steel and Ni-Ti ceramic bracket (n.1) **Duration:** MD **Discontinuation/Appliance replacement:** MD	**Skin involvement:** MD **Skin location:** MD **Eye involvement:** MD **Systemic involvement**: MD	**Oral mucosal lesions, hypo-/dys-geusia +/− hypo-/dys-geusia +/− hyposmia, dysestesia:** none **Oral macroscopic features:** MD **Number:** MD **Distribution**: MD **Location:** MD **Oral microscopic features:** MD **Time to onset:** MD	**Allergy test(s):** patch test **Timing of the allergic test performed:** 4 months after the beginning of the OT **Evidence of allergy to orthodontic materials:** To (Ni): n.2 PS (1M/1F) **DD**: MD **Definitive oral diagnosis:** MD **Definitive extra-oral diagnosis:** MD **Pharmacological Therapy**: MD **Resolution/Progression:** MD
Counts, A.L. 2002 J Orofac Orthop. Case-report [24] Critical No Funding	**Sample:** n.1 **Mean Age/Range:** 12 y.o. **Gender ratio:** 1F **History of allergies or hypersensitivity:** MD **Previous allergy test:** MD **Other comorbidities**: marginal gingivitis **Ongoing pharmacological** **therapies:** MD **Piercing:** Ear P after 2 months of OT	**Orthodontic Treatment Appliance:** FA (RPE with soldered bilateral tongue cribs, followed by TPA) **Type of brackets:** MD **Type of archwire:** MD **Orthodontic material:** MD **Duration:** MD **Discontinuation/Appliance replacement:** Supragingival biofilm removal Removal of the second TPA with soldered lateral tongue cribs, and substitution without Ni-Ti wires	**Skin involvement:** Unspecified rash **Skin location:** abdomen **Eye involvement:** MD **Systemic involvement**: MD	**Oral mucosal lesions, hypo-/dys-geusia +/− hypo-/dys-geusia +/− hyposmia, dysestesia:** gingival hyperplasia, redness **Oral macroscopic features:** MD **Number:** MD **Distribution**: MD **Location:** gingiva **Oral microscopic features:** MD **Time to onset:** MD	**Allergy test(s):** patch test **Timing of the allergic test performed:** MD **Evidence of allergy to orthodontic materials:** To (Ni): n.1 PS (1F) **DD**: MD **Definitive oral diagnosis:** MD **Definitive extra-oral diagnosis:** dermatitis **Pharmacological Therapy**: MD **Resolution/Progression:** Redness and hypertrophy had greatly diminished after three months
Ehrnrooth, M. 2009 Angle Orthod. Case-report [25] Critical No Funding	**Sample:** n.1 **Mean Age/Range:** 34 y.o. **Gender ratio:** 1 F **History of allergies or hypersensitivity:** Suspected nickel allergy **Previous allergy test:** MD **Other comorbidities**: hay fever **Ongoing pharmacological therapies:** antihistamine medication **Piercing:** MD	**Orthodontic Treatment Appliance:** FA + RPE Expanor screw Hyrax II (Dentaurum 1.000 SS; 1.003 SS/ remanium; 1.002 SS, Ni content 8–10%; Dentaurum Ispringen, Germany); Four bands (Trimline 18/8 SS; Ormco Corp, Orange, Calif) **Type of brackets:** MD **Type of archwire:** MD **Orthodontic material:** Stainless steel **Duration:** MD **Discontinuation/Appliance replacement:** RPE removal	**Skin involvement:** Itchy papular erythema; Itchy Unspecified rash and vesicles. **Skin location:** from check to the chest; neck **Eye involvement:** Redness Itching Tearing **Systemic involvement**: MD	**Oral mucosal lesions, hypo-/dys-geusia +/− hypo-/dys-geusia +/− hyposmia, dysestesia:** None **Oral macroscopic features:** MD **Number:** MD **Distribution**: MD **Location:** MD **Oral microscopic features:** MD **Time to onset:** MD	**Allergy test(s):** patch tests with 5% Ni sulphate, 1% cobalt chloride, and 1% Pa-chloride. **Timing of the allergic test performed:** MD **Evidence of allergy to orthodontic materials:** To (Ni): n.1 PS (1F) To (Co): n.1 PS (1F) To (Pa): n.1 PS (1F) **DD**: MD **Definitive oral diagnosis:** MD **Definitive extra-oral diagnosis:** MD **Pharmacological Therapy**: MD **Resolution/Progression:** Healing after 4–5 days after RPE removal
Feilzer, A.J. 2008 Contact Dermatitis Case-report [26] Critical No Funding	**Sample:** n.1 **Mean Age/Range:** 35 y.o. **Gender ratio:** 1F **History of allergies or hypersensitivity:** Sensitization to dust and some mascara types; PS to fragrance mix II, Cr and Ni. **Previous allergy test:** Patch test with the European standard series and the cosmetic series **Other comorbidities**: Von Willebrand’s disease **Ongoing pharmacological** **therapies:** MD **Piercing:** Ear P	**Orthodontic Treatment Appliance:** FA (contention retainer wires) **Type of brackets:** MD **Type of archwire:** MD **Orthodontic material:** Ni 8%; Fe 75%; Mn 1%; Cr 16%. **Duration:** MD **Discontinuation/Appliance replacement:** substitution of retainer wires with a thin plastic splint	**Skin involvement:** N/D **Skin location:** Face **Eye involvement:** MD **Systemic involvement**: MD	**Oral mucosal lesions, hypo-/dys-geusia +/− hypo-/dys-geusia +/− hyposmia, dysestesia:** MD **Oral macroscopic features:** MD **Number:** MD **Distribution**: MD **Location:** MD **Oral microscopic features:** MD **Time to onset:** MD	**Allergy test(s):** blood test (6 months after removal of the splint); Lymphocyte proliferation test **Timing of the allergic test performed:** MD **Evidence of allergy to orthodontic materials:** To (Ni): n.1 PS (1F) To (Hg): n.1 PS (1F) To (Cr): n.1 PS (1F) **DD**: MD **Definitive oral diagnosis:** MD **Definitive extra-oral diagnosis:** Eczema **Pharmacological Therapy**: MD **Resolution/Progression:** Improvement of the eczema 4 weeks after the removal of the retainer wires
Fors, M. 2012 Contact Dermatitis Cross-sectional study [12] Moderate No Funding	**Sample:** n.405 **Mean Age/Range:** 14–23,4 y.o. **Gender ratio:** 15M/390F **History of allergies or hypersensitivity:** MD **Previous allergy test:** patch test **Other comorbidities**: MD **Ongoing pharmacological** **therapies:** MD **Piercing:** n.1228 p (85M/1143F) Before OT: n.875 (56M/819F) After OT: n.353 (29M/324F)	**Orthodontic Treatment Appliance:** FA with and without extra-oral traction + RME **Type of brackets:** MD **Type of archwire:** Lingual arches **Orthodontic material:** Stainless steel Acrylic resin **Duration:** MD **Discontinuation/Appliance replacement:** MD	**Skin involvement:** MD **Skin location:** MD **Eye involvement:** MD **Systemic involvement**: MD	**Oral mucosal lesions, hypo-/dys-geusia +/− hypo-/dys-geusia +/− hyposmia, dysestesia:** MD **Oral macroscopic features:** MD **Number:** MD **Distribution**: MD **Location:** MD **Oral microscopic features:** MD **Time to onset:** MD	**Allergy test(s):** patch test **Timing of the allergic test performed:** MD **Evidence of orthodontic materials allergy:** To (Ni): n.204PS (3M/201F) not P: n.17 (9M/8F) P before OT: n.156 (3M/153F) P after OT: n.28 (0M/28F) **DD**: MD **Definitive oral diagnosis:** MD **Definitive extra-oral diagnosis:** MD **Pharmacological Therapy**: MD **Resolution/Progression:** MD
Giancotti, A. 2011 Eur J Paediatr Dent. Case-report [27] Moderate Funding No	**Sample:** n. 1 **Mean Age/Range:** 10 y.o. **Gender ratio:** 1M **History of allergies or hypersensitivity:** Ni-allergy **Previous allergy test:** specific medical tests **Other comorbidities**: MD **Ongoing pharmacological** **therapies:** MD **Piercing:** MD	**Orthodontic Treatment Appliance:** CAs (Essix) **Type of brackets:** MD **Type of archwire:** MD **Orthodontic material:** copolyester **Duration:** 12 weeks **Discontinuation/Appliance replacement:** MD	**Skin involvement:** MD **Skin location:** MD **Eye involvement:** MD **Systemic involvement**: MD	**Oral mucosal lesions, hypo-/dys-geusia +/− hypo-/dys-geusia +/− hyposmia, dysestesia:** MD **Oral macroscopic features:** MD **Number:** MD **Distribution**: MD **Location:** MD **Oral microscopic features:** MD **Time to onset:** MD	**Allergy test(s):** MD **Timing of the allergic test performed:** MD **Evidence of allergy to orthodontic materials:** none **DD**: MD **Definitive oral diagnosis:** MD **Definitive extra-oral diagnosis:** MD **Pharmacological Therapy**: MD **Resolution/Progression:** MD
Haraguchi, S. 2014 Angle Orthod. Case-report [28] Serious Funding No	**Sample:** n. 1 **Mean Age/Range:** 28 y.o. **Gender ratio:** 1F **History of allergies or hypersensitivity:** hypersensitivity to Ni, Co, and Cr. **Previous allergy test:** patch test **Other comorbidities**: MD **Ongoing pharmacological** **therapies:** MD **Piercing:** MD	**Orthodontic Treatment Appliance:** FA (preadjusted edgewise appliances + Ti microscrews—Dual-Top Anchor System, 8.0 mm in length, 1.6 mm in diameter; Jeil Medical Corporation, Seoul, Korea—Acrylic posterior bite blocks) **Type of brackets:** 0.022-inch slot preadjusted edgewise brackets (Equilibrium ti, Dentaurum, Ispringen, Germany) **Type of archwire:** Ni-free beta Ti 0.016-inch, 0.017-inch, 0.025-inch (intrusion arch) wires (CNA BetaIII Archwires, Ortho Organizers Inc, Carlsbad, Calif) **Orthodontic material:** Pure Ti **Duration:** 26 months 2 years of retention **Discontinuation/Appliance replacement:** MD	**Skin involvement:** MD **Skin location:** MD **Eye involvement:** MD **Systemic involvement**: MD	**Oral mucosal lesions, hypo-/dys-geusia +/− hypo-/dys-geusia +/− hyposmia, dysestesia:** MD **Oral macroscopic features:** MD **Number:** MD **Distribution**: MD **Location:** MD **Oral microscopic features:** MD **Time to onset:** MD	**Allergy test(s):** MD **Timing of the allergic test performed:** MD **Evidence of allergy to orthodontic materials:** To (Ni): n.1 PS (1F) To (Cr): n.1 PS (1F) To (Co): n.1 PS (1F) **DD**: MD **Definitive oral diagnosis:** MD **Definitive extra-oral diagnosis:** MD **Pharmacological Therapy**: MD **Resolution/Progression:** MD
Janson, G.R. 1998 Am J Orthod Dentofacial Orthop. Case-control study [29] Critical No Funding	**Sample:** n. 48 **Mean Age/Range:** 12–31 y.o. **Gender ratio:** 9M/39F **History of allergies or hypersensitivity:** N/D **Previous allergy test:** MD **Other comorbidities**: MD **Ongoing pharmacological** **therapies:** MD **Piercing:** N/D	**Orthodontic Treatment Appliance:** FA **Type of brackets:** MD **Type of archwire:** MD **Orthodontic material:** Ni-Cr (AISI 302, 18%Cr, 8%Ni) **Duration:** MD **Discontinuation/Appliance replacement:** MD	**Skin involvement:** MD **Skin location:** MD **Eye involvement:** MD **Systemic involvement**: MD	**Oral mucosal lesions, hypo-/dys-geusia +/− hypo-/dys-geusia +/− hyposmia, dysestesia:** MD **Oral macroscopic features:** MD **Number:** MD **Distribution**: MD **Location:** MD **Oral microscopic features:** MD **Time to onset:** MD	**Allergy test(s):** Ni patch test and 5% Ni sulfate in white petrolatum. **Timing of the allergic test performed:** during OT (n.66); after OT (n.44) **Evidence of allergy to orthodontic materials:** To (Ni): n.48 PS (9M/39F) **DD**: MD **Definitive oral diagnosis:** MD **Definitive extra-oral diagnosis:** MD **Pharmacological Therapy**: MD **Resolution/Progression:** MD
Johansson, K. 2011 Contact Dermatitis Prospective study [30] Serious No Funding	**Sample:** n.13 **Mean Age/Range:** 14.7 y.o.; 10–39 y.o. **Gender ratio:** MD **History of allergies or hypersensitivity:** MD **Previous allergy test** First patch test: To (Ni): n.7 PS **Other comorbidities**: MD **Ongoing pharmacological** **therapies:** MD **Piercing:** n.35 (86%)	**Orthodontic Treatment Appliance:** FA **Type of brackets:** MD **Type of archwire:** Elastic archwires Stiff archwires **Orthodontic material:** Stainless steel with 8–12% Ni; elastic archwires with a high Ni content (50% or more); stiff archwires with a low Ni content. **Duration:** MD **Discontinuation/Appliance replacement:** MD	**Skin involvement:** MD **Skin location:** MD **Eye involvement:** MD **Systemic involvement**: MD	**Oral mucosal lesions, hypo-/dys-geusia +/− hypo-/dys-geusia +/− hyposmia, dysestesia:** None **Oral macroscopic features:** MD **Number:** MD **Distribution**: MD **Location:** MD **Oral microscopic features:** MD **Time to onset:** MD	**Allergy test(s):** patch tests with Ni sulfate (5% in petrolatum) **Timing of the allergic test performed:** First test: before OT Second test: 12.4 months later first patch test **Evidence of allergy to orthodontic materials:** Second patch test: To (Ni): n.6 PS Subjects with more piercings had positive patch tests significantly more often than subjects with one or no piercings. **DD**: MD **Definitive oral diagnosis:** MD **Definitive extra-oral diagnosis:** MD **Pharmacological Therapy**: MD **Resolution/Progression:** MD
Kalimo, M. 2004 J Eur Acad Dermatol Venereol Observational cross-sectional study [31] Critical Funding: No	**Sample:** n. 30 **Mean Age/Range:** 22 y.o. **Gender ratio:** 3M/27F **History of allergies** **or hypersensitivity:** MD **Previous allergy test:** MD **Other comorbidities**: MD **Ongoing pharmacological** **therapies:** MD **Piercing:** n.52 Before FA: n.20 After FA: n.24 After RA(S): n.8	**Orthodontic Treatment Appliance:** FA: N/D RA: N/D **Type of brackets:** MD **Type of archwire:** MD **Orthodontic material:** Nickel and other metals **Duration:** 2 years (6 months–8 years) **Discontinuation/Appliance replacement:** MD	**Skin involvement:** MD **Skin location:** MD **Eye involvement:** MD **Systemic involvement**: MD	**Oral mucosal lesions, hypo-/dys-geusia +/− hypo-/dys-geusia +/− hyposmia, dysestesia:** MD **Oral macroscopic features:** MD **Number:** MD **Distribution**: MD **Location:** MD **Oral microscopic features:** MD **Time to onset:** MD	**Allergy test(s):** patch test **Timing of the allergic test performed:** MD **Evidence of orthodontic materials allergy:** To (Ni): n.30 PS (3M/27F) not P + FA: n.4 (3M/1F) P before FA: n.11 (0M/11F) P after FA: n.10 (0M/10F) P after RA(S): n.5 (0M/5F) **DD**: MD **Definitive oral diagnosis:** MD **Definitive extra-oral diagnosis:** MD **Pharmacological Therapy**: MD **Resolution/Progression:** MD
Kelso, J.M. 2007 Ann Allergy Asthma Immunol. Case-report [32] Critical No Funding	**Sample:** n.1 **Mean Age/Range:** 49 y.o. **Gender ratio:** 1F **History of allergies or hypersensitivity:** MD **Previous allergy test:** MD **Other comorbidities**: MD **Ongoing pharmacological** **therapies:** MD **Piercing:** MD	**Orthodontic Treatment Appliance:** CAs **Type of brackets:** MD **Type of archwire:** MD **Orthodontic material:** 95–98% natural rubber latex, 0–1.0% sulfur, 0–1.0% zinc oxide, 1–2.0% polymeric hindered phenol, and 0–1.0% dithiocarbonate derivative. **Duration:** MD **Discontinuation/Appliance replacement:** Rubber bands discontinuation	**Skin involvement:** MD **Skin location:** MD **Eye involvement:** MD **Systemic involvement**: MD	**Oral mucosal lesions, hypo-/dys-geusia +/− hypo-/dys-geusia +/− hyposmia, dysestesia:** Sore **Oral macroscopic features:** Vesicles **Number:** MD **Distribution**: MD **Location:** generalized oral cavity gum **Oral microscopic features:** MD **Time to onset:** two weeks later band application	**Allergy test(s):** patch test panel of 23 common sensitizing agents (carba mix, black rubber mix, mercaptobenzothiazole, mercapto mix, and thiuram mix, a piece of a latex surgical glove, a piece of a latex toy balloon, and one of the orthodontic rubber bands) **Timing of the allergic test performed:** MD **Evidence of allergy to orthodontic materials:** To (Ni): n.1 PS (1F) To orthodontic rubber band: n.1 PS (1F) **DD**: MD **Definitive oral diagnosis:** Allergic contact stomatitis **Definitive extra-oral diagnosis:** Delayed-type hypersensitivity reactions to latex **Pharmacological Therapy**: Oral antibiotics **Resolution/Progression:** Stomatitis resolved within 2 weeks after the band was discontinued
Kerosuo, H. 1996 Am J Orthod Dentofacial Orthop. Case-control study [33] Moderate No Funding	**Sample:** n.84 **Mean Age/Range:** 14–18 y.o. **Gender ratio:** 2M/82F **History of allergies or hypersensitivity:** MD **Previous allergy test:** MD **Other comorbidities**: MD **Ongoing pharmacological** **therapies:** MD **Piercing:** N/D	**Orthodontic Treatment Appliance:** FA: N/D RA: N/D **Type of brackets:** MD **Type of archwire:** MD **Orthodontic material:** Ni **Duration:** 3–40 months **Discontinuation/Appliance replacement:** MD	**Skin involvement:** MD **Skin location:** MD **Eye involvement:** MD **Systemic involvement**: MD	**Oral mucosal lesions, hypo-/dys-geusia +/− hypo-/dys-geusia +/− hyposmia, dysestesia:** MD **Oral macroscopic features:** MD **Number:** MD **Distribution**: MD **Location:** MD **Oral microscopic features:** MD **Time to onset:** MD	**Allergy test(s):** patch test (Finn chambers on Scanpor Surgical Tape) with 5% Ni-sulphate in petrolatum **Timing of the allergic test performed:** MD **Evidence of allergy to orthodontic materials:** To (Ni): n.84 PS (2M/82F) p before FA: n.50 (0M/50F) p before Quad helix: n.6 (0M/6F) p before Headgear: n.19 (1M/18F) p after Quad helix: n.4 (0M/4F) p after Headgear: n.5 (1M/4F) **DD**: MD **Definitive oral diagnosis:** MD **Definitive extra-oral diagnosis:** MD **Pharmacological Therapy**: MD **Resolution/Progression:** MD
Kerosuo, H. 1997 Contact Dermatitis. Case-report [34] Critical Funding	**Sample:** n.1 **Mean Age/Range:** 14 y.o. **Gender ratio:** 1M **History of allergies or hypersensitivity:** atopy **Previous allergy test:** MD **Other comorbidities**: MD **Ongoing pharmacological** **therapies:** MD **Piercing:** MD	**Orthodontic Treatment Appliance:** RA (Extraoral face bow + metal molar bands) **Type of brackets:** MD **Type of archwire:** MD **Orthodontic material:** Stainless steel 18% Cr and 8% Ni **Duration:** MD **Discontinuation/Appliance replacement:** Device discontinuation and intraoral bands removal	**Skin involvement:** scaling and fissures; crusted lesions and vesicles. **Skin location:** Palm and soles Scalp Abdomen Around lips Legs **Eye involvement:** MD **Systemic involvement**: MD	**Oral mucosal lesions, hypo-/dys-geusia +/− hypo-/dys-geusia +/− hyposmia, dysestesia:** MD **Oral macroscopic features:** MD **Number:** MD **Distribution**: MD **Location:** MD **Oral microscopic features:** MD **Time to onset:** 4 weeks later started OT	**Allergy test(s):** patch test The face bow was tested by the dimethylglyoxime spot test for nickel **Timing of the allergic test performed:** MD **Evidence of allergy to orthodontic materials:** PS to Ni PS to Co **DD**: MD **Definitive oral diagnosis:** MD **Definitive extra-oral diagnosis:** eczema; dermatitis. **Pharmacological Therapy**: local medication for eczema **Resolution/Progression:** Worsening of eczema after 7 weeks. Healing of dermatitis and stomatitis after the face bow was discontinued and bands were removed.
Kolokitha, O.E. 2009 Angle Orthod. Case-report [35] Critical No Funding	**Sample:** n.1 **Mean Age/Range:** 27 y.o. **Gender ratio:** 1F **History of allergies or hypersensitivity:** No **Previous allergy test:** MD **Other comorbidities**: MD **Ongoing pharmacological** **therapies:** MD **Piercing:** MD	**Orthodontic Treatment Appliance:** FA (with Coil spring) + RA (Hawley retainer) **Type of brackets:** (0.022 0.028-in) metal buttons with steel ligature ties with fabricated bent loops on impacted canine (13 and 23) **Type of archwire:** 0.018 Bioforce Sentalloy archwires, (GAC, Bohemia, NY, USA); 0.025 NiTi archwire **Orthodontic material:** Ni-Ti **Duration:** 3 years **Discontinuation/Appliance replacement:** removal of the attached button of the upper maxillary left canine. Replacement of metal brackets with ceramic brackets and coated Ni-Ti archwires	**Skin involvement:** Unspecified rash; reactions, redness, irritation, itching, soreness, fissures, and desquamation. **Skin location:** Face **Eye involvement:** MD **Systemic involvement**: MD	**Oral mucosal lesions, hypo-/dys-geusia +/− hypo-/dys-geusia +/− hyposmia, dysestesia:** erythema **Oral macroscopic features:** MD **Number:** MD **Distribution**: Diffuse **Location:** MD **Oral microscopic features:** MD **Time to onset:** 4 days after the surgical exposure of the impacted canine for bonding the metal buttons with steel ligature with fabricated bent loops (4 months after the initial bonding of other teeth)	**Allergy test(s):** patch test **Timing of the allergic test performed:** MD **Evidence of allergy to orthodontic materials:** To (Ni): n.1 PS To (Thiomersal): n.1 PS **DD**: MD **Definitive oral diagnosis:** MD **Definitive extra-oral diagnosis:** Eczema; Urticaria; Allergic contact dermatitis **Pharmacological Therapy**: N/D **Resolution/Progression:** Improvement after the removal of the attached button of the upper maxillary left canine. Resolution after 7 months
Mancuso, G. 2002 Contact Dermatitis Case-report [36] Critical No Funding	**Sample:** n.1 **Mean Age/Range:** 13 y.o. **Gender ratio:** 1F **History of allergies or hypersensitivity:** allergy to Ni and eyeshadow **Previous allergy test:** patch test **Other comorbidities**: MD **Ongoing pharmacological** **therapies:** topical ophthalmic therapy (failed) **Piercing:** MD	**Orthodontic Treatment Appliance:** RA **Type of brackets:** MD **Type of archwire:** MD **Orthodontic material:** Steel containing 10–13% Ni and 16–19% Cr **Duration:** MD **Discontinuation/Appliance replacement:** low Ni diet; RA Substitution of the with new Ni-free FA	**Skin involvement:** symmetrical erythema and edema. **Skin location:** cheeks; upper and lower eyelids. **Eye involvement: C**onjunctival hyperemia of both eyes. **Systemic involvement**: MD	**Oral mucosal lesions, hypo-/dys-geusia +/− hypo-/dys-geusia +/− hyposmia, dysestesia:** none **Oral macroscopic features:** MD **Number:** MD **Distribution**: MD **Location:** MD **Oral microscopic features:** MD **Time to onset:** MD	**Allergy test(s):** patch test; prick test **Timing of the allergic test performed:** MD **Evidence of allergy to orthodontic materials:** To (Ni): n.1 PS (1F) **DD**: MD **Definitive oral diagnosis:** MD **Definitive extra-oral diagnosis:** MD **Pharmacological Therapy**: MD **Resolution/Progression:** healing of eyes involvement within 2 weeks after removal, with relapse of dermatitis following reinsertion of the appliance. No more lesions after appliance replacement
Maspero C. 2014 Minerva Stomatol. Case-control [37] Moderate Funding: No	**Sample:** n. 80 **Mean Age/Range:** 10–15 y.o. **Gender ratio:** 36 M/60F **History of allergies or hypersensitivity:** To (Ni): n.16 (6M,10F) PS before OT, 94% positive family history **Previous allergy test:** patch test (n.16 6M/10F) **Other comorbidities**: MD **Ongoing pharmacological** **therapies:** MD **Piercing:** MD	**Orthodontic Treatment Appliance:** RA (Frankel function regulator + FA (RPE) **Type of brackets:** MD **Type of archwire:** MD **Orthodontic material:** Ti **Duration:** MD **Discontinuation/Appliance replacement:** MD	**Skin involvement:** none **Skin location:** MD **Eye involvement:** MD **Systemic involvement**: MD	**Oral mucosal lesions, hypo-/dys-geusia +/− hypo-/dys-geusia +/− hyposmia, dysestesia:** none **Oral macroscopic features:** MD **Number:** MD **Distribution**: MD **Location:** MD **Oral microscopic features:** MD **Time to onset:** MD	**Allergy test(s):** patch test **Timing of the allergic test performed:** MD **Evidence of allergy to orthodontic materials:** To (Ni): n.80 (30M/50F) PS after OT, 98% positive family history In patients PS → regression of the symptoms **DD**: MD **Definitive oral diagnosis:** MD **Definitive extra-oral diagnosis:** MD **Pharmacological Therapy**: MD **Resolution/Progression:** MD
Menezes, L.M. 2004 Am J Orthod Dentofacial Orthop. Prospective study [1] Serious No Funding	**Sample:** n. 38 **Mean Age/Range:** 9–25 y.o. **Gender ratio:** 17M/21F **History of allergies or hypersensitivity:** MD **Previous allergy test:** patch test To (Co): n.0 PS To (Cu): n.0 PS To(Cr): n.8 PS (5M/3F) To (Ir): n.0 PS To (Mn): n.3 PS (0M/3F) To (Mo): n.0 PS To (Ni): n.8 PS (2M/6F) To (Ti): n.2 PS (2M/0F) **Other comorbidities**: MD **Ongoing pharmacological** **therapies:** MD **Piercing:** MD	**Orthodontic Treatment Appliance:** FA **Type of brackets:** MD **Type of archwire:** MD **Orthodontic material:** Co; Cu; Cr; Ir; Mn; Mo Ni; Ti **Duration:** MD **Discontinuation/ Appliance replacement:** MD	**Skin involvement:** MD **Skin location:** MD **Eye involvement:** MD **Systemic involvement**: MD	**Oral mucosal lesions, hypo-/dys-geusia +/− hypo-/dys-geusia +/− hyposmia, dysestesia:** MD **Oral macroscopic features:** MD **Number:** MD **Distribution**: MD **Location:** MD **Oral microscopic features:** MD **Time to onset:** MD	**Allergy test(s):** patch tests with 2% Co-chloride, 5% Cu- sulfate, 0.5% potassium dichromate, 2% Ir-sulfate, 1% Mn-chloride, 1% Mo- salt, 5% Ni-sulfate, and 1% Ti-oxide. **Timing of the allergic test performed:** before OT + 2 months after FA placement **Evidence of allergy to orthodontic materials:** To (Co): n.0 PS To (Cu): n.0 PS To (Cr): n.10 PS (6M/4F) To (Ir): n.0 PS To (Mn): n.1 PS (0M/1F) To (Mo): n.1 PS (1M/0F) To (Ni): n.8 PS (1M/7F) To (Ti): n.0 PS **DD**: MD **Definitive oral diagnosis:** MD **Definitive extra-oral diagnosis:** MD **Pharmacological Therapy**: N/D topical treatment **Resolution/Progression:** MD
Pantuzo, M.C.G. 2007 Braz Oral Res. Prospective study [47] Serious No Funding	**Sample**: n.33 **Mean Age/Range:** 11–30 y.o. **Gender ratio:** MD **History of allergies or hypersensitivity:** To (Ni): n.16 PS **Previous allergy test:** patch test to Ni **Other comorbidities:** MD **Ongoing pharmacological** **therapies:** none **Piercing:** MD	**Orthodontic Treatment Appliance:** FA **Type of brackets:** Morelli brackets (Dental Morelli Ltd.a.—Sorocaba, SP, Brazil) **Type of archwire:** MD **Orthodontic material:** Ni **Duration:** MD **Discontinuation/Appliance replacement:** MD	**Skin involvement:** MD **Skin location:** MD **Eye involvement:** MD **Systemic involvement:** MD	**Oral mucosal lesions, hypo-/dys-geusia +/− hypo-/dys-geusia +/− hyposmia, dysestesia:** MD **Oral macroscopic features:** MD **Number:** MD **Distribution:** MD **Location:** MD **Oral microscopic features:** MD **Time to onset:** MD	**Allergy test(s):** patch test to Ni with a similar composition of a conventional bracket; patch test to Ni with a similar composition of Ni-free bracket **Timing of the allergic test performed:** MD **Evidence of allergy to orthodontic materials:** To (Ni): n.12 PS to only conventional bracket n.5 PS to both conventional and Ni-free bracket **DD:** MD **Definitive oral diagnosis:** MD **Definitive extra-oral diagnosis:** MD **Pharmacological Therapy:** MD **Resolution/Progression:** MD
Paschaei, N. 2024 J Clin Med [46] Prospective study Deutsche Gesellschaft für Umweltzahnmedizin (DEGUZ) e.V and the Charité—Universitätsmedizin Berlin and the German Research Foundation	**Sample**: n.6 **Mean Age/Range:** N/D **Gender ratio:** 2F/4M **History of allergies or hypersensitivity:** MD **Previous allergy test:** blood test; Lymphocyte proliferation test **Other comorbidities:** none **Ongoing pharmacological** **therapies:** MD **Piercing:** MD	**Orthodontic Treatment Appliance:** FA **Type of brackets:** metal brackets (3M, Dentaurum, Orthana, and Forestadent) **Type of archwire:** metal archwire (3M, Dentaurum, Orthana, and Forestadent) **Orthodontic material:** MD **Duration:** MD **Discontinuation/Appliance replacement:** MD	**Skin involvement:** MD **Skin location:** MD **Eye involvement:** MD **Systemic involvement:** MD	**Oral mucosal lesions, hypo-/dys-geusia +/− hypo-/dys-geusia +/− hyposmia, dysestesia:** discomfort of the oral mucosa **Oral macroscopic features:** MD **Number:** MD **Distribution:** generalized oral mucosa **Location:** MD **Oral microscopic features:** MD **Time to onset:** MD	**Allergy test(s):** blood test; Lymphocyte proliferation test **Timing of the allergic test performed:** 21 days after FA placement (n.4) 21 days after FA removal (n.1) 1 day before FA placement (n.1) **Evidence of allergy to orthodontic materials:** To (Ni): n.5 PS (1F/4M) To (Pd): n.1 PS (1F) **DD:** MD **Definitive oral diagnosis:** MD **Definitive extra-oral diagnosis:** MD **Pharmacological Therapy:** MD **Resolution/Progression:** MD
Pazzini, C.A. 2012 Angle Orthod. RCT [4] High CAPES	**Sample:** n.42 **Mean Age/Range:** 10–45 y.o. **Gender ratio:** 14M/28F **History of allergies or hypersensitivity:** MD **Previous allergy test:** MD **Other comorbidities**: MD **Ongoing pharmacological** **therapies:** prophylaxis with bicarbonate spray **Piercing:** MD	**Orthodontic Treatment Appliance:** FA **Type of brackets:** MD **Type of archwire:** MD **Orthodontic material:** FA with conventional appliances: 16–20% Cr, 8–13% Ni, 2–3% Mo or n.21 FA with nickel-free appliances: more than 18% Cr, 0.2–4% Ni, 3.5% Mo. **Duration:** MD **Discontinuation/Appliance replacement:** MD	**Skin involvement:** MD **Skin location:** MD **Eye involvement:** MD **Systemic involvement**: MD	**Oral mucosal lesions, hypo-/dys-geusia +/− hypo-/dys-geusia +/− hyposmia, dysestesia:** MD **Oral macroscopic features:** MD **Number:** MD **Distribution**: MD **Location:** MD **Oral microscopic features:** MD **Time to onset:** MD	**Allergy test(s):** Patch test with 5% Ni-sulfate **Timing of the allergic test performed:** MD **Evidence of allergy to orthodontic materials:** To (Ni): n.42 PS (14M/28F) **DD**: MD **Definitive oral diagnosis:** MD **Definitive extra-oral diagnosis:** MD **Pharmacological Therapy**: MD **Resolution/Progression:** MD
Pazzini, C.A. 2016 Am J Orthod Dentofacial Orthop RCT [38] High No funding	**Sample:** n.42 **Mean Age/Range:** 10–45 y.o. **Gender ratio:** 14M/28F **History of allergies or hypersensitivity:** MD **Previous allergy test:** MD **Other comorbidities**: MD **Ongoing pharmacological** **therapies:** prophylaxis with bicarbonate spray **Piercing:** MD	**Orthodontic Treatment Appliance:** FAs **Type of brackets:** MD **Type of archwire:** MD **Orthodontic material:** FA with conventional appliances: 16– 20% Cr, 8–13% Ni, 2–3% Mo or n.21 FA with nickel-free appliances: more than 18% Cr, 0.2–4% Ni, 3.5% Mo. **Duration:** MD **Discontinuation/Appliance replacement:** MD	**Skin involvement:** MD **Skin location:** MD **Eye involvement:** MD **Systemic involvement**: MD	**Oral mucosal lesions, hypo-/dys-geusia +/− hypo-/dys-geusia +/− hyposmia, dysestesia:** MD **Oral macroscopic features:** MD **Number:** MD **Distribution**: MD **Location:** MD **Oral microscopic features:** MD **Time to onset:** MD	**Allergy test(s):** patch test with 5% Ni-sulfate **Timing of the allergic test performed:** MD **Evidence of allergy to orthodontic materials:** To (Ni): n.42 PS (14M/28F) **DD**: MD **Definitive oral diagnosis:** MD **Definitive extra-oral diagnosis:** MD **Pharmacological Therapy**: MD **Resolution/Progression:** MD
Pigatto, P.D. 2004 Contact Dermatitis Case-report [39] Critical No Funding	**Sample:** n.1 **Mean Age/Range:** 14 y.o. **Gender ratio:** 1F **History of allergies or hypersensitivity:** Atopy **Previous allergy test:** MD **Other comorbidities**: MD **Ongoing pharmacological** **therapies:** MD **Piercing:** MD	**Orthodontic Treatment Appliance:** FA **Type of brackets:** MD **Type of archwire:** MD **Orthodontic material:** MD **Duration:** MD **Discontinuation/Appliance replacement:** Appliance removal	**Skin involvement:** erythema, edema with vesiculation and crusting **Skin location:** Ears; Face; Neck; Scalp; Upper arms (flexures); Hands (dorsal); Wrists **Eye involvement:** MD **Systemic involvement**: MD	**Oral mucosal lesions, hypo-/dys-geusia +/− hypo-/dys-geusia +/− hyposmia, dysestesia:** None **Oral macroscopic features:** MD **Number:** MD **Distribution**: MD **Location:** MD **Oral microscopic features:** MD **Time to onset:** MD	**Allergy test(s):** patch test **Timing of the allergic test performed:** MD **Evidence of allergy to orthodontic materials:** To (Ni): n.1 PS (1F) **DD**: MD **Definitive oral diagnosis:** MD **Definitive extra-oral diagnosis:** MD **Pharmacological Therapy**: MD **Resolution/Progression:** Healing of cutaneous lesions with no scarring, within 6 months
Saglam, A.M. 2004 J Contemp Dent Pract. Cross-sectional study [40] RA(s)erate Funding: No	**Sample:** n. 16 **Mean Age/Range:** 14.32 y.o.; 11–20 y.o. **Gender ratio:** 4M/12F **History of allergies or hypersensitivity:** n.3 **Previous allergy test:** MD **Other comorbidities**: MD **Ongoing pharmacological** **therapies:** MD **Piercing:** MD	**Orthodontic Treatment Appliance:** FA (Edge-wise) **Type of brackets:** MD **Type of archwire:** MD **Orthodontic material:** Stainless steel Ni-Ti Co **Duration:** MD **Discontinuation/Appliance replacement:** MD	**Skin involvement:** MD **Skin location:** MD **Eye involvement:** MD **Systemic involvement**: MD	**Oral mucosal lesions, hypo-/dys-geusia +/− hypo-/dys-geusia +/− hyposmia, dysestesia:** MD **Oral macroscopic features:** MD **Number:** MD **Distribution**: MD **Location:** MD **Oral microscopic features:** MD **Time to onset:** MD	**Allergy test(s):** Ni patch test, 5% nickel sulphate, and 1% cobalt in white petrolatum. **Timing of the allergic test performed:** MD **Evidence of allergy to orthodontic materials:** To (Ni): n.8 PS (0M/8F) To (Co): n.8 PS (4M/4F) **DD**: MD **Definitive oral diagnosis:** MD **Definitive extra-oral diagnosis:** MD **Pharmacological Therapy**: MD **Resolution/Progression:** MD
Shargill, I. 2015 Dent Update. Case-report [41] Critical No Funding	**Sample:** n. 1 **Mean Age/Range:** 13 y.o. **Gender ratio:** 1F **History of allergies or hypersensitivity:** MD **Previous allergy test:** MD **Other comorbidities**: mild asthma **Ongoing pharmacological** **therapies:** salbutamol inhaler and Becotide **Piercing:** MD	**Orthodontic Treatment Appliance:** FA (with intermaxillary elastics) and RA (Headgear) **Type of brackets:** MD **Type of archwire:** MD **Orthodontic material:** MD **Duration:** 2–2.5 years **Discontinuation/Appliance replacement:** latex components of fixed appliances were removed; reduction of the use of archwires with high Ni content and omission of Ni active components (Ni-Ti closing springs headgear wear was terminated)	**Skin involvement:** edema and crusts **Skin location:** lips **Eye involvement:** MD **Systemic involvement**: MD	**Oral mucosal lesions, hypo-/dys-geusia +/− hypo-/dys-geusia +/− hyposmia, dysestesia:** Gingival enlargement **Oral macroscopic features:** MD **Number:** MD **Distribution**: MD **Location:** upper and lower gums **Oral microscopic features:** MD **Time to onset:** One day after orthodontic adjustments.	**Allergy test(s):** N/D allergy test to Ni and latex Blood test **Timing of the allergic test performed:** MD **Evidence of allergy to orthodontic materials:** none **DD**: trauma-induced edema following dental extractions; delayed type IV hypersensitivity reaction to latex; type IV cell-mediated delayed hypersensitivity reaction to Ni **Definitive oral diagnosis:** MD **Definitive extra-oral diagnosis:** pressure or contact urticaria **Pharmacological Therapy**: Anti-histamine (oral Loratadine 10 mg the night before orthodontic appointments and 10 mg the day of the procedure) **Resolution/Progression:** healing after pharmacological treatment
Tammaro, A. 2015 Eur. Ann. Allergy Clin. Immunol. Case-report [42] Critical No Funding	**Sample:** n.1 **Mean Age/Range:** 12 y.o. **Gender ratio:** 1M **History of allergies or hypersensitivity:** MD **Previous allergy test:** MD **Other comorbidities**: MD **Ongoing pharmacological** **therapies:** MD **Piercing:** MD	**Orthodontic Treatment Appliance:** RA (adjustable dynamic protraction facemask-Ormco–Sybron) **Type of brackets:** MD **Type of archwire:** MD **Orthodontic material:** Ni-sulphate **Duration:** MD **Discontinuation/Appliance replacement:** removal of facial mask	**Skin involvement:** Erythema papular itchy lesions **Skin location:** perioral **Eye involvement:** MD **Systemic involvement**: MD	**Oral mucosal lesions, hypo-/dys-geusia +/− hypo-/dys-geusia +/− hyposmia, dysestesia:** MD **Oral macroscopic features:** MD **Number:** MD **Distribution**: MD **Location:** MD **Oral microscopic features:** MD **Time to onset:** MD	**Allergy test(s):** patch test with standard series SIDAPA containing the following haptens: K-Dichromate; Rosin; Epoxy Resin; Formaldehyde Resin; Euxil 400; Neomycin Sulphate; Fragrance Mix; Ni-Sulphate; Mercaptobenzothiazole Paraphenylendiamine; Co-Chloride; Balsam of Peru; Thiuram Mix; Benzocaine; Lanolin Alcohols; Parabens; Vaseline; Scattered Yellow; Scattered Blue; Hydroquinone **Timing of the allergic test performed:** MD **Evidence of allergy to orthodontic materials:** To (Ni): n.1 PS (1M) To fragrance mix: n.1 PS (1M) **DD**: MD **Definitive oral diagnosis:** MD **Definitive extra-oral diagnosis:** MD **Pharmacological Therapy**: local corticosteroids and antihistamines **Resolution/Progression:** Worsening of the perioral lesion after the topical application of corticosteroids; healing of the skin lesions after the removal of the facial mask.
Veien, N.K. 1994 Contact Dermatitis Case-series [43] Critical No Funding	**Sample:** n.5 **Mean Age/Range:** 14.6 y.o.; 13–16 y.o. **Gender ratio:** 5F **History of allergies or hypersensitivity:** MD **Previous allergy test:** MD **Other comorbidities:** MD **Ongoing pharmacological** **therapies:** MD **Piercing:** Ear P	**Orthodontic Treatment Appliance:** FA **Type of brackets:** MD **Type of archwire:** MD **Orthodontic material:** steel containing 17–19% Cr and 10–13% Ni; Acrylic and steel wire containing 10–27% Cr and 12–34% Ni; Wires containing 18% Ni and 8% Co. Wires containing 10–13% Ni and 17–19% Cr. **Duration:** MD **Discontinuation/Appliance replacement:** **Case 1:** OT was discontinued 1 year later (n.1) **Case 2:** Metal wires were replaced with acrylics (n.1) **Case 3:** OT was not discontinued **Case 4:** OT was discontinued (n.1) **Case 5:** OT was discontinued (n.1)	**Skin involvement:** 1 case: N/D 2 case: N/D 3 case: N/D 4 case: N/D 5 case: pruritus and dryness of the lips **Skin location:** Fingers; Perioral area; Lips; Eyelids **Eye involvement:** MD **Systemic involvement:** MD	**Oral mucosal lesions, hypo-/dys-geusia +/− hypo-/dys-geusia +/− hyposmia, dysestesia:** **To (Ni):** Case 5: Pruritus and discomfort of the buccal mucosa. **Oral macroscopic features:** Erosions; vesicles **Number:** MD **Distribution:** MD **Location:** buccal mucosa **Oral microscopic features:** MD **Time to onset:** MD	**Allergy test(s):** patch test (n.5) prick test (n.4) oral challenge test (n.4) **Timing of the allergic test performed:** MD **Evidence of allergy to orthodontic materials:** Tot. PS at patch test: n.2 Tot. PS at prick test: n. 1 Tot. PS at oral challenge test: n. 4 Case 1: To (Cr): n.1 PS (1F) (patch test and oral challenge test) and to gress pollen (prick test) Case 2: To (Cr): n.1 PS (1F) (oral challenge test) and PN to patch test and prick test Case 3: To (Ni): n.1 PS (1F) (oral challenge test) and PN to patch test and prick test Case 4: To (Co): n.1 PS (1F) (oral challenge test) and PN to patch test and prick test Case 5: To (Ni): n.1 PS (1F) (patch test) and PN to oral challenge test **DD:** MD **Definitive oral diagnosis:** (undefined) Stomatitis **Definitive extra-oral diagnosis:** Dermatitis; Eczema **Pharmacological Therapy:** MD **Resolution/Progression:** **Case 1:** Fingers dermatitis 2 months after device removal (n.1). **Case 2:** Dermatitis cleared 1 month after being replaced with acrylic removal (n.1). **Case 3:** Eczema on the fingers is controlled well by following a low-Ni diet. removal (n.1) **Case 4:** Dermatitis cleared after some weeks after device removal (n.1) **Case 5:** Symptoms disappeared 1 month after device removal (n.1)
Velásquez, D. 2010 Allergol Immunopathol (Madr). Case-report [44] Serious Funding No	**Sample:** n.1 **Mean Age/Range:** 23 y.o. **Gender ratio:** 1F **History of allergies or hypersensitivity:** allergy to penicillin **Previous allergy test:** MD **Other comorbidities**: MD **Ongoing pharmacological** **therapies:** MD **Piercing:** MD	**Orthodontic Treatment Appliance:** MD **Type of brackets:** MD **Type of archwire:** MD **Orthodontic material:** MD **Duration:** MD **Discontinuation/Appliance replacement:** MD	**Skin involvement:** erythema, fissurations and scaly itching lesions; itching and a burning sensation. **Skin location:** dorsum of fingers and hands; Lips. **Eye involvement:** MD **Systemic involvement**: MD	**Oral mucosal lesions, hypo-/dys-geusia +/− hypo-/dys-geusia +/− hyposmia, dysestesia:** itching and a burning sensation **Oral macroscopic features:** MD **Number:** MD **Distribution**: MD **Location:** generalized oral cavity **Oral microscopic features:** MD **Time to onset:** MD	**Allergy test(s):** patch testing was performed with the Spanish standard series (TRUEtests, ALK-Abello’, Madrid), metals and the acrylates series (including Mn) **Timing of the allergic test performed:** MD **Evidence of allergy to orthodontic materials:** To (Mn): n.1 PS (1F) **DD**: MD **Definitive oral diagnosis:** allergic contact stomatitis **Definitive extra-oral diagnosis:** allergic dermatitis contact **Pharmacological Therapy**: local and systemic corticosteroids **Resolution/Progression:** Healing after the cycle of oral corticosteroids
Zigante, M. 2020 Prog Orthod Observational cross-sectional study [45,48,49] Moderate Croatian Science Foundation	**Sample:** n.37 **Mean Age/Range:** 11–45 years old **Gender ratio:** 6M/31F **History of allergies or hypersensitivity:** Contact hypersensitivity (n.19 PS to Ni; n.1 PS to Ti); Contact hypersensitivity to metal (n.6 PS to Ni; n.1 PS to Ti) Contact hypersensitivity to imitation jewellery (n.14 PS to Ni; n.1 PS to Ti) **Previous allergy test:** MD **Other comorbidities**: No **Ongoing pharmacological** **therapies:** MD **Piercing:** n.11 (n.8 PS to Ni; n.3 PS to Ti)	**Orthodontic Treatment Appliance:** FA **Type of brackets:** metallic brackets (Ortho Classic, USA) **Type of archwire:** Archwires (GAC International, Japan) **Orthodontic material:** Ni-Ti; Ni; Ti **Duration:** minimum of 6 weeks, maximum of 1 year **Discontinuation/Appliance replacement:** MD	**Skin involvement:** **To (Ni)** Swelling of face n.5 PS **To (Ti)** Swelling of face n.5 PS **Skin location:** Face **Eye involvement:** **To (Ti)** Watery eyes n.1 PS **To (Ni)** Watery eyes n.7 PS **Systemic involvement**: **To (Ti)** Vertigo n.3 PS	**Oral mucosal lesions, hypo-/dys-geusia +/− hypo-/dys-geusia +/− hyposmia, dysestesia:** **To (Ni)** Swelling of tongue: n.5 PS Weakened sense of taste: n.1 PS Weakened sense of smell: n.1 PS **To (Ti)** Swelling of tongue: n.5 PS Oral burning: n.2 PS Dysgeusia: n.3 PS Weakened sense of taste: n.2 PS Weakened sense of smell: n.2 PS **Oral macroscopic features:** MD **Number:** MD **Distribution**: MD **Location:** tongue **Oral microscopic features:** MD **Time to onset:** MD	**Allergy test(s):** Epicutaneous patch test to nickel sulfate, titanium, titanium dioxide, titanium oxalate, and titanium nitride with petrolatum used as control **Timing of the allergic test performed:** 2–24 months after FA placement **Evidence of orthodontic materials allergy:** To (Ti): n.5 PS (1M/4F) To (Ni): n.32 PS (1M/26F) **DD**: MD **Definitive oral diagnosis:** N/D **Definitive extra-oral diagnosis:** MD **Pharmacological Therapy**: MD **Resolution/Progression:** MD

Acronyms: “M”, men; “F”, females; “n”, number; “y.o.”, years old; “PS”, positive sensibilization; “NS”, negative sensibilization; “Ni”, nickel; “Ti”, titanium; “Co”, cobalt; “Pa”, palladium; “Mo”, molybdenium; “Mn”, manganese; “Ir”, iron; “Cr”, chromium; “Cu”, copper; “Hg”, mercury; “MD”, missing data; “N/D”, not defined; “DD”, differential diagnosis; “OT”, orthodontic treatment; “p”, piercing; “FA(s)”, fixed-orthodontic appliance(s); “RA(s)”, removable-orthodontic appliance(s); “CA(s)”, clear aligner(s); “EOA”, extra-oral appliance; “B”, basophils; “E”, eosinophils; “TPA”, trans palatal arch; “RPE”, rapid palatal expansion; “RME”, rapid maxillary expansion.

## Data Availability

Data are available in the Scopus, MEDLINE/PubMed, and Web of Science databases and the PROSPERO register.

## Supplementary Materials

The following supporting information can be downloaded at: https://www.mdpi.com/article/10.3390/jcm14134766/s1, File S1: Quality Assessment.
