# Supplementary material for: Hypersensitivity in Orthodontics: A Systematic Review of Oral and Extra-Oral Reactions
Source: J Clin Med. 2025 Jul 5;14(13):4766. doi: 10.3390/jcm14134766 (PMC12250706; doi:10.3390/jcm14134766)
Supplement: Supplementary file 1 [file jcm-14-04766-s001.zip › jcm-3726521-supplementary.pdf]

Hypersensitivity in Orthodontics: A Systematic Review of Oral and Extra-Oral Reactions

Alessandra Amato[1], Stefano Martina[2], Giuseppina De Benedetto[2], Ambrosina Michelotti[1], Massimo Amato[2,*,†] and Federica Di Spirito[2,*,†]

[1] Department of Neuroscience, Reproductive Science and Dentistry, University of Naples Federico II,
80131 Naples, Italy; aaleamato@gmail.com (A.A.); ambrosina.michelotti@unina.it (A.M.)
[2] Department of Medicine, Surgery and Dentistry, University of Salerno, Via S. Allende, 84081 Baronissi, Salerno, Italy; smartina@unisa.it (S.M.); giusydb15@gmail.com (G.D.B.)
* Correspondence: mamato@unisa.it (M.A.); fdispirito@unisa.it (F.D.S.)
† These authors contributed equally to this work

Supplementary File S1: Quality assessment

Table S1. Risk of Bias of non-randomized studies of exposures included in the present systematic review.

Study	Confound ing	Selection of Participants	Classification of Interventions	Deviations from Intended Interventions	Bias Due to Missing Data	Measurement of Outcomes	Selection of Reported Result	Overall Risk judgment
Awosika et al., 2017 [23]	Moderate	Low	Critical	Low	Low	Moderate	Low	Critical
Bass et al., 1993 [24]	Low	Moderate	Critical	Critical	Moderate	Moderate	Low	Critical
Counts et al., 2002 [25]	Low	Low	Low	Critical	Low	Moderate	Low	Critical
Ehrnrooth et al, 2009 [26]	Low	Low	Serious	Critical	Low	Moderate	Low	Critical
Feilzer et al., 2008 [27]	Moderate	Low	Low	Critical	Low	Serious	Low	Critical
Fors et al., 2012 [13]	Low	Low	Low	Low	Low	Moderate	Low	Moderate
Giancotti et al., 2011 [28]	Moderate	Low	Low	Low	Low	Low	Low	Moderate
Haraguchi et al.,2014 [29]	Moderate	Low	Low	Serious	Moderate	Moderate	Low	Serious
Janson et al., 1998 [30]	Low	Critical	Low	Serious	Low	Low	Moderate	Critical
Johansson et al., 2011 [31]	Low	Serious	Moderate	Low	Moderate	Moderate	Low	Serious
Kalimo et al., 2004 [32]	Moderate	Low	Low	Moderate	Low	Low	Critical	Critical
Kelso et al., 2007 [33]	Low	Low	Moderate	Critical	Low	Moderate	Low	Critical
Kerosuo et al., 1996 [34]	Low	Low	Moderate	Moderate	Low	Moderate	Moderate	Moderate
Kerosuo et al., 1997 [35]	Low	Moderate	Moderate	Critical	Moderate	Moderate	Low	Critical
Kolokitha et al., 2009 [36]	Moderate	Moderate	Serious	Critical	Moderate	Moderate	Moderate	Critical
Mancuso et al., 2002 [37]	Moderate	Low	Moderate	Critical	Low	Moderate	Moderate	Critical
Maspero et al., 2014 [38]	Low	Low	Moderate	Low	Moderate	Moderate	Moderate	Moderate
Menezes et al., 2004 [1]	Low	Moderate	Low	Moderate	Moderate	Moderate	Serious	Serious
Pantuzo et al., 2007 [48]	Moderate	Low	Moderate	Serious	Moderate	Low	Low	Serious
Paschaei et al., 2024 [47]	Low	Low	Moderate	Low	Moderate	Moderate	Moderate	Moderate
Pigatto et al., 2004 [40]	Low	Low	Moderate	Critical	Low	Low	Low	Critical
Saglam et al., 2004 [41]	Moderate	Moderate	Low	Moderate	Moderate	Low	Low	Moderate
Shargill et al., 2015 [42]	Serious	Low	Low	Critical	Low	Moderate	Low	Critical
Tammaro et al., 2015 [43]	Low	Low	Moderate	Critical	Low	Moderate	Moderate	Critical
Veien et al., 1994 [44]	Moderate	Low	Moderate	Critical	Moderate	Moderate	Low	Critical
Velásquez et al., 2010 [45]	Low	Low	Low	Serious	Moderate	Low	Low	Serious
Zigante et al., 2020 [45]	Low	Moderate	Low	Moderate	Moderate	Low	Low	Moderate
Zigante et al., 2022 [49]	Low	Moderate	Low	Moderate	Moderate	Low	Low	Moderate
Zigante et al., 2020 [50]	Low	Moderate	Low	Moderate	Moderate	Low	Low	Moderate

Table S2. Risk of Bias judgment of randomized clinical trials included in the present systematic review.

Study	Randomization process	Effect of assignment to intervention	Effect of adhering to intervention	Missing outcome data	Measurement of the outcome	Selection of the reported result	Overall Risk judgment
Pazzini et al., 2012 [4]	Low	Some concerns	Some concerns	High	Low	Some concerns	High
Pazzini et al., 2016 [39]	Low	Some concerns	Some concerns	High	Low	Some concerns	High
